# Observation of critical magnetic behavior in 2D carbon based composites

**DOI:** 10.1039/c9na00663j

**Published:** 2020-01-09

**Authors:** Vineeta Shukla

**Affiliations:** Nuclear Condensed Matter Physics Laboratory, Department of Physics, Indian Institute of Technology Kharagpur-721302 India vineetashukla2@yahoo.com.in

## Abstract

Two dimensional (2D) carbonaceous materials such as graphene and its derivatives, *e.g.*, graphdiyne, have enormous potential possibilities in major fields of scientific research. Theoretically, it has been proposed that the perfect atomic lattice arrangement of these materials is responsible for their outstanding physical and chemical properties, and also for their poor magnetic properties. Experimentally, it is difficult to obtain a perfect atomic lattice of carbon atoms due to the appearance of structural disorder. This structural disorder is generated during the growth or synthesis of carbon-related materials. Investigations of structural disorder reveal that it can offer both advantages and disadvantages depending on the application. For instance, disorder reduces the thermal and mechanical stability, and deteriorates the performance of 2D carbon-based electronic devices. The most interesting effect of structural disorder can be seen in the field of magnetism. Disorder not only creates magnetic ordering within 2D carbon materials but also influences the local electronic structure, which opens the door for future spintronic devices. Although various studies on the disorder induced magnetism of 2D carbon materials are available in the literature, some parts of the above field have still not been fully exploited. This review presents existing work for the future development of 2D carbon-based devices.

## Abstract 2

This review article discusses the magnetic interactions attained by 2D carbon related materials.

## Graphical abstract

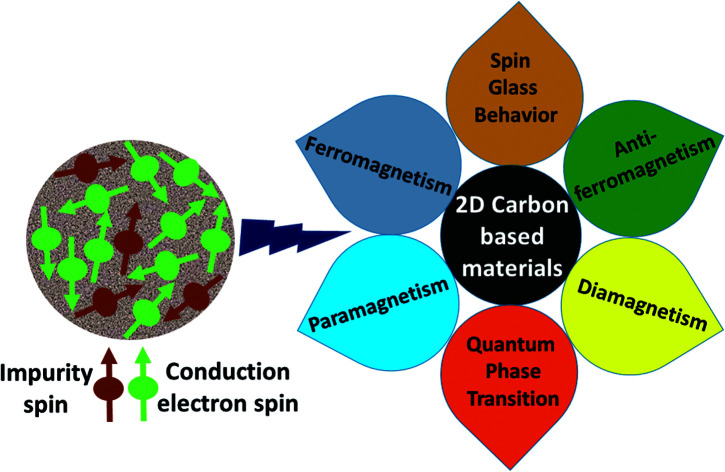

## 1 Introduction

**Fig. 1:**
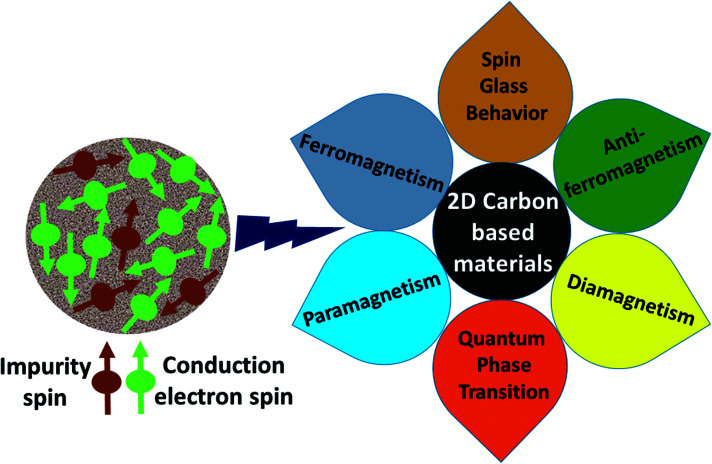
A schematic representation of magnetic interactions attained by 2D carbon related materials.

Carbon (a p-block element) is the plentiful sixth element in the universe with two well-known allotropes: graphite and diamond. σ and π bonds bind the carbon atoms to form a molecule. Thus, the number and nature of the bonds determine the properties and geometries of the carbon allotropes. After the discovery of the 21^st^ century’s first 2D carbon material, known as graphene, it is anticipated that graphene could form the basis of other carbon allotropes like 0D fullerenes and 1D carbon nanotube materials. Graphene is an sp^2^-bonded one-atom-thick sheet with a honeycomb crystal lattice. It has extraordinary properties such as high theoretical specific surface area (2630 m^2^ g^−1^), high thermal conductivity (1500–2500 W m^−1^ K^−1^), superior intrinsic mobility (∼200 000 cm^2^ V^−1^ s^−1^ at a carrier density of 1012 cm^−2^) even at room temperature, high mechanical stability (tensile strength ∼130.5 GPa), excellent optical transmittance (∼98% observed for red light), extremely high electrical conductivity (10^6^ Ω^−1^ cm^−1^), *etc.*, because it exhibits Dirac-like electron excitations which result in unusual properties.^1,2^ Quantum mechanically, it is anticipated that confinement of electrons in single layer carbon materials increases transport phenomena, resulting in the quantum Hall effect^3^ which favors a non-zero Berry phase of graphene (*i.e.*, a topological phase). Various attractive phenomena like the Casimir effect,^4^ and the quantum magneto-optical Faraday and Kerr effects^5^ can be seen in graphene due to the linear energy dispersion relation. How to make a versatile and cost-effective material which can be tuned according to requirements has always been the subject of puzzlement in the fabrication of materials for energy storage, biomedicine and electronic devices. In the science community, graphene has been accepted as one of the most desirable materials due to its light weight, unusual properties and ease of synthesis. In modern technology, magnetic materials are crucial for various applications such as spintronics, biomedicine, magnetic (bio) separation, microwave absorption, *etc.* Spintronics is an emerging area of condensed matter physics and is of particular interest in the field of quantum and neuromorphic computing. Spintronics is quite similar to electronics, except it uses electron spin degrees of freedom instead of the electrical charge of the electron used in electronics, and that is why it is also known as spin electronics. Spin is an inherent property of particles. Thus, the use of electron spin degrees of freedom provides a logic bit, which increases the data processing speed, energy efficiencies and integration densities of the information storage and logic operations and also decreases the power consumption. Spin-polarization can be achieved in magnetic materials. In general, magnetic materials are metals in which imbalance between unpaired spin-up (↑) and spin-down (↓) electrons leads to ferromagnetism. Magnetism occurs in both the d- and the f-block elements of the periodic table, *e.g.*, the transition metals Fe (3d^6^4s^2^), Co (3d^7^4s^2^), and Ni (3d^8^4s^2^) contain partially filled d orbitals and are renowned ferromagnets at room temperature. Super-exchange interactions are responsible for magnetic coupling between the spins of the metal ions and can be expressed by the isotropic Heisenberg Hamiltonian: 
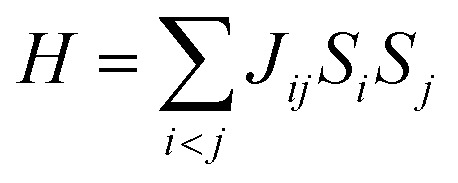
, where *J*_*ij*_ is the coupling constant between the *i*^th^ spin and the *j*^th^ spin. In comparison with metals, semiconductors have long relaxation times and lengths which make them ideal for spintronics applications. In this area, Heusler alloys and diluted magnetic semiconductors (DMSs) are commonly used to make spin based devices such as spin-light emitting diodes, spin injection and spin-transfer torque devices, spin field-effect transistors, large magneto-resistance spin valves, *etc.*^6^ The problem with Heusler alloys and DMSs is the formation of complex interfaces due to high carrier-doping levels. Thus, for superior storage devices, we aim to achieve ferromagnetism in DMSs by reducing the doping level in the semiconductor materials. Apart from this, heavy weight, high cost and corrosion related problems also hinder the use of metal/semiconductor materials. Now let us think about the second period elements in the periodic table. The p-block elements possess several attractive properties, including biocompatibility, low density and plasticity, which are desirable for several potential applications. Moreover, light element based composites demonstrate weak spin–orbit and hyper-fine splitting phenomena which are accountable for the spin relaxation process and decoherence of electron spins. Nonetheless, magnetism is not common in light p-block elements like carbon, although it can occur in various molecular structures. The lack of d or f shell electrons in p-block elements makes them magnetically neutral, which is a major issue for condensed matter scientists. Therefore, d^0^ magnetism has attracted attention from physicists. Resulting from structural defects such as vacancies, adatoms are a well-known example of d^0^ magnetism. It is noteworthy that a suitable combination of structural defects and host can make graphene an active magnetic material. Magnetic centers within graphene can even be modulated by varying the defect concentration, since the coordination number in the 2D lattice arrangement can be reduced by low concentrations of defects that weaken the coupling. This can be achieved with artificial defects. Nevertheless, increasing the number of defects cannot be considered an agreeable solution. This is because a high concentration of defects may perturb the crystal structure and result in unwanted physical properties. Another way of inducing d^0^ magnetism is the doping of materials with a p-type 2D host matrix. Some researchers have observed that p-type impurities like magnetic ions can induce magnetic moments in some semiconductors. Therefore, d^0^ magnetism can be achieved by doping, which controls the magnetism in the p-type host. It was seen that dopants with smaller radius, compared with the host material, result in higher on-site stability of the localized spins, similar to d- or f-type^7^ dopants. Thus, the atomic radius is an important factor in tuning d^0^ magnetism by controlling the wavefunction tails of localized electrons. Therefore, two-dimensional d^0^ magnetism can be achieved even by first-row element adatoms in graphene.^8^ Previous reports on the possible magnetism of carbon structures show controversy due to the poor reproducibility of experimental results for carbon materials. But the situation has improved over the last few years because modification can be achieved by chemical treatment, grafting of defects and vacancies, anchoring of magnetic impurities, *etc.*, which all lead to magnetism in carbon-based materials. Observations reveal that magnetism can also be tuned by an external gate voltage (*V*_g_) that influences the Fermi energy. A recently published paper showed that gate-tunable magnetism could even be attained by putting C adatoms in the graphene structure.^9^ When the impurity spins couple with the conduction electron spins, as shown in Fig. 1, different kinds of magnetism, like paramagnetism, ferromagnetism, antiferromagnetism or spin glass type behavior, can be obtained in 2D carbon based composites according to the coupling of the spins within the material. Now the question is why 2D carbon based composites are more desirable than the wide range of other 2D materials such as transition metal dichalcogenides, for example MoS_2_, VS_2_, WS_2_, TiSe_2_, TiS_2_, NbS_2_, *etc.*,^10^ even though these materials have remarkable and desirable electronic, optical and mechanical properties. Also, transition metal dichalcogenides (TMDCs) have potential applications in energy storage devices including hydrogen evolution devices, light-emitting devices, sodium-ion batteries, and various other energy conversion applications. In comparison to TMDCs, 2D carbon based composites can be considered a better choice because the conductivity of 2D carbon based composites varies from the metallic to the semiconductor state, and their bio-compatibility and ease of synthesis make them appealing for exciting applications in broad scientific areas. For example, good microwave materials could be obtained by balancing the relative complex permittivity and permeability because the dielectric loss and magnetic loss have complementary relations.^11^ The intrinsic surface impedance in relation to the complex permittivity (*ε* = *ε*′ + i*ε*′′) and permeability (*μ* = *μ*′ + j*μ*′′) for a given medium can be written as^12^1
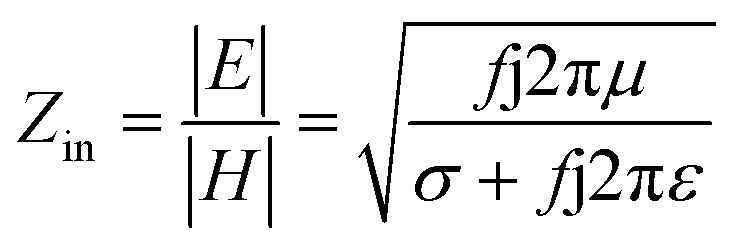
Here *σ* is the conductivity of the material and *f* is the frequency. Microwave absorption is represented in terms of reflection loss:2
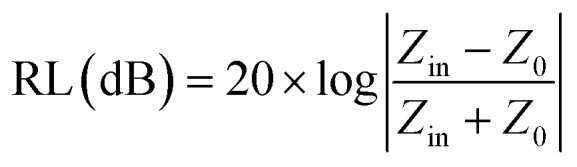
Here *Z*_0_ is the impedance of air, and *Z*_in_ is the input impedance of the absorber. Minimum reflection loss (RL_min_) occurs when the impedance of free space and the impedance of the composite match. The ideal impedance matching condition is *Z*_in_ = *Z*_0_ = 377 Ω. Thus, tuning of the electrical and magnetic properties is crucial, and depending on the application this can easily be achieved in 2D carbon materials rather than in TMDCs.

The present review provides a brief overview of current research into the magnetic behavior of graphene based nanostructures. Firstly, the theoretical aspects according to the mean-field Hubbard Hamiltonian are briefly presented, and secondly, the obtained experimental results in this field are summarised. In addition, we discuss experimental tools for studying the magnetic nature of 2D carbon based composites. It is anticipated that the present review would be helpful in understanding the critical magnetic behavior of 2D carbon based composites and would pave the way for the fabrication of memory, spintronics and other energy storage devices.

## 2 Carbonaceous materials

### 2.1 Graphene

**Fig. 2:**
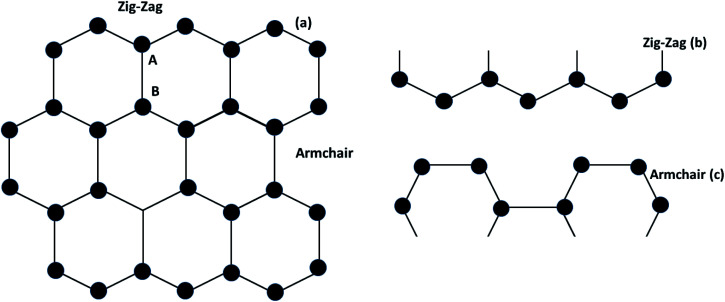
(a) Graphene structure, (b) zig-zag type- and (c) armchair types of graphene.

**Fig. 3:**
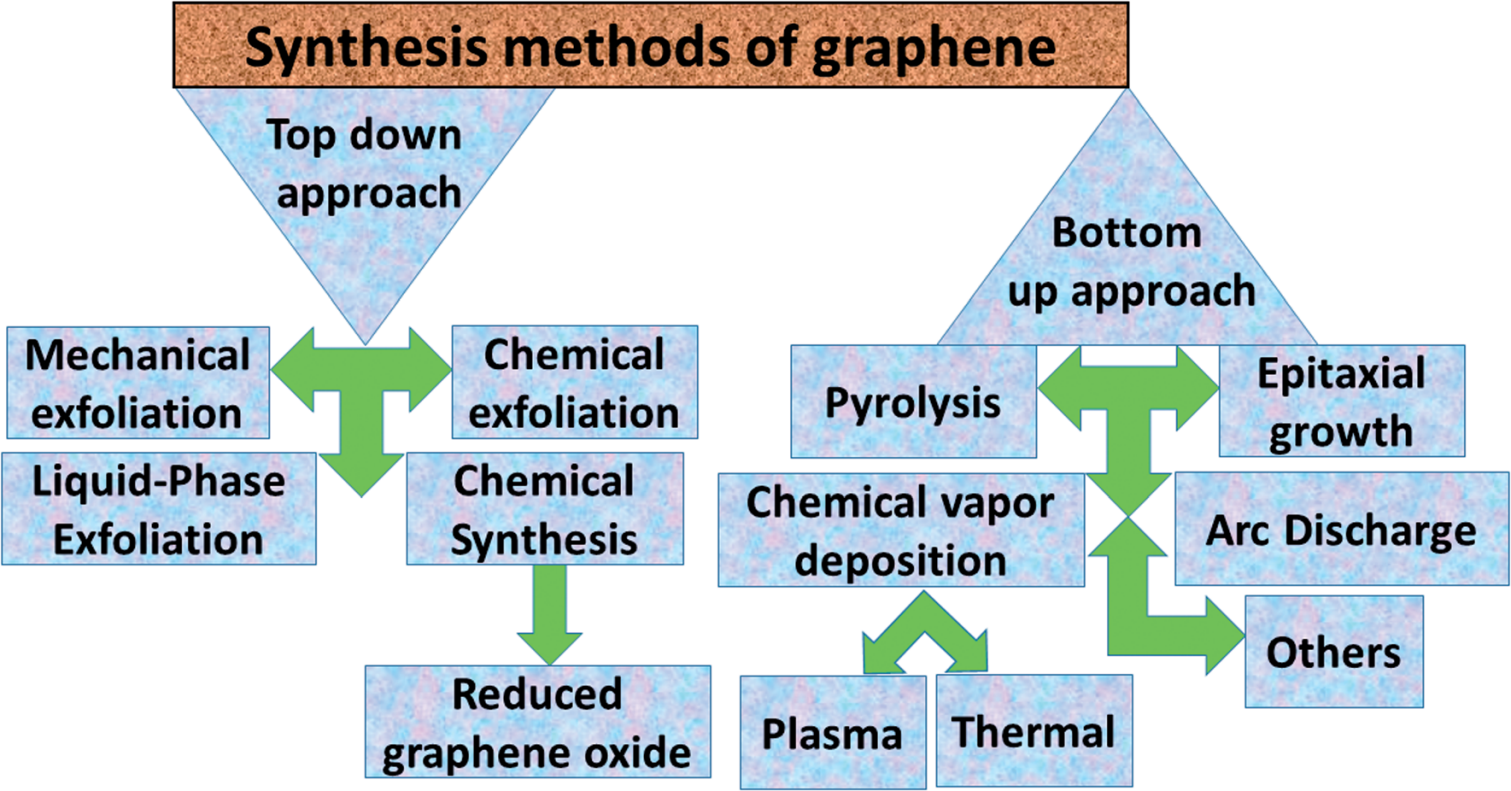
Abbreviated description of the synthesis methods for graphene.

Graphene contains simple sp^2^ hybridized carbon atoms in a honeycomb structure. Its Hamiltonian (*H*) is described by the Dirac equation in relativistic quantum mechanics in terms of the mass-less Dirac fermion. *H* is expressed by the following Weyl equation:

3 *H* = *v*_F_*σp*

where *v*_F_ and *p* are the Fermi velocity and the momentum, respectively. *σ* is the pseudo-spin. Thus, the electronic structure of pristine graphene consists of two Dirac cones located at the *K* and *K*′ points in the Brillouin zone. The positions of the Dirac cones are accountable for graphene’s unique electronic structure and make it a semi-metal. This electronic structure with linear dispersion controls most of the physical properties of the material. Graphene has wide applications in printed electronics and conductive coatings owing to its extraordinary properties as explained in an earlier section. Graphene was first discovered in 2004 by Novoselov and Geim, who used Scotch tape to obtain the graphene sheet through the mechanical exfoliation method. If the graphene sheet is cut, zig-zag and armchair edges are produced as depicted in Fig. 2(a–c). These edges greatly influence the electronic structure of graphene. The geometry of the edges determines the effect of the edges on the physical properties of graphene materials. For instance, longer zig-zag edges (more than 3–4 units) are recognized to be highly localized and independent of the shape of the edges (regular or irregular).^13^ It was reported that zig-zag edges have nonbonding π-electron states with localized spins in the zig-zag region, simply called edge states, but that armchair edges do not possess these edge states. The presence of edge states is an outcome of the split symmetry of the pseudo spin at the zig-zag edge. On the other hand, this pseudo spin symmetry remains preserved in the armchair edges.^14^ It is expected that edge irregularities, even defects, located at the bounding edges in 2D carbon materials contribute to the total edge state magnetism. Theoretically, graphene has a bipartite lattice of sublattices A and B and intrinsically possesses zero magnetic moments. However, the second law of thermodynamics demonstrates the presence of some disorder in the crystal lattice system. Even quantum mechanics predict the zero point energy at *T* = 0 K. If this is true, then there is a possibility of there being disorder in graphene in the form of vacancies, defects or other imperfections. It has been demonstrated that structural disorder always remains in crystalline graphene if it arises during the growth of the material. Therefore the different aspect of graphene magnetism has been proposed by the researchers over the last decades rather searching is continued. There are many factors like gate voltage, doping, the interactions of atoms, *etc.* that affect the electronic and magnetic properties of graphene. Among them, structural disorder, such as defects, impurities or patterning, and chemical treatment of the material are known to be excellent sources of local magnetic moments. The concentration of structural disorder depends on the fabrication method. Several methods have been reported to synthesize graphene. They all belong to two major categories: (1) bottom-up approach and (2) top-down approach, as shown in Fig. 3. These different approaches offer graphene of different size and quality according to the specific application. Some top-down approaches are mechanical exfoliation, chemical exfoliation, chemical reduction, *etc.*, and each has some benefits and some drawbacks. The large scale production of graphene is not possible by mechanical exfoliation, while chemical oxidation disturbs the electronic structure of graphene, which limits its application in device fabrication even though it is considered favorable for some applications like electromagnetic interference shielding.^12^ Also, as a result of chemical treatment, some organic species and defects remain on the zig-zag or basal planes of graphene. These structure peculiarities create localized states which break the pseudo-spin symmetry of graphene and give rise to localized spins. Bottom-up approaches include CVD and epitaxial growth, which can produce large-size graphene with tunable thickness. The major hindrance of using these techniques is the dependency on the substrate, which can limit the dimensions and increase the cost. Thus it is anticipated that it cannot fulfil the requirements for commercial consumption of elevated-quality graphene.^15,16,17^ Additionally, the pseudo-spin symmetry is conserved in this case due to the lack of defects/vacancies and adatoms, which is a major problem for memory devices and spin-based devices. Tucek *et al.* have shown that the controlled sp^3^ functionalization of graphene to make zig-zag conjugated sp^2^ carbon chains can provide a suitable matrix for strong ferromagnetic ordering up to room temperature. This is because of the combination of super exchange interactions and contributions from itinerant π-electrons.^18^ From this, it can be concluded that the synthesis method plays an important role in tuning d^0^ magnetism.

### 2.2 Graphene oxide

**Fig. 4:**
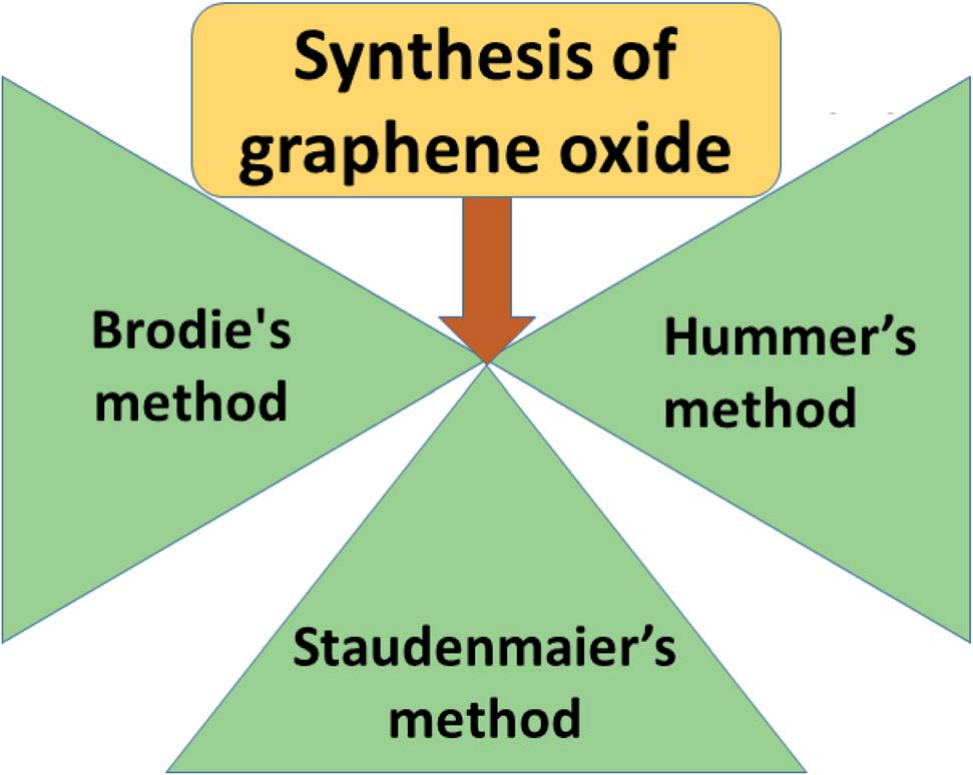
Fabrication methods for graphene oxide.

**Fig. 5:**
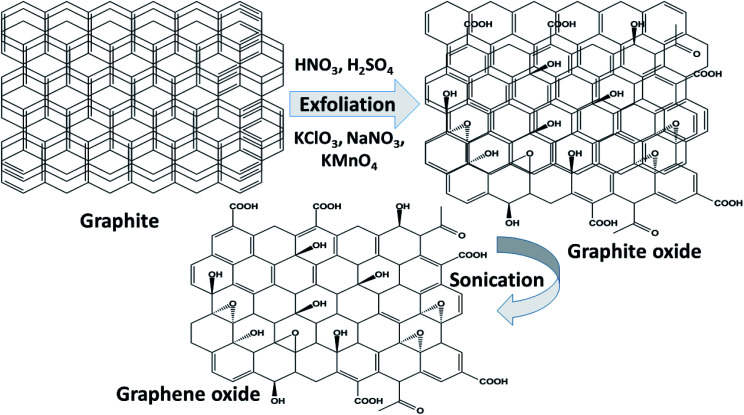
A schematic representation of the synthesis of GO by chemical methods.

Graphene oxide (GO) is a typical graphene derivative. Moreover, GO is well recognized as an oxygen functionalized highly disordered graphene sheet which is not stoichiometric. GO can exist as a monolayer or as a few stacked layers. It is proposed that the energetically favorable places for oxygen-containing groups such as –OH (hydroxyl), –O– (epoxy), –COOH (carboxyl) and –C=O (carbonyl) groups are the basal planes and edges of graphene sheets. The presence of these functionalities makes it hydrophilic, unlike graphene, which is hydrophobic. This property of GO makes it easy to disperse in most solvents and increases its applicability in various applications. Compared to fluorographene, GO is less stable and can be reversibly transformed into the unoxidized state by chemical or thermal reduction. Also, GO has a high porosity, high surface area, excellent mechanical strength, and high chemical stability. Fig. 4 shows the synthesis methods for GO. Brodie (1859) first carried out the oxidation of graphite, using potassium chlorate (KClO_3_) and fuming nitric acid (HNO_3_). Later, the above method became known as the Brodie method. In 1898, Staudenmaier improved this protocol by adding H_2_SO_4_ along with fuming nitric acid. In 1937, Hofmann made a change to this method by using concentrated HNO_3_ in place of fuming nitric acid, concentrated H_2_SO_4_ and KClO_3_. Nevertheless, these methods were hazardous to humans due to the generation of toxic gases like ClO_2_, which is explosive. In 1958, Hummer introduced a simple method for the oxidation of graphite in the presence of concentrated sulphuric acid (H_2_SO_4_), KMnO_4_ and NaNO_3_, known as Hummer’s method. In all the above methods, intercalation of compounds such as KClO_3_, KMnO_4_ and NaNO_3_ weakens the van der Waals forces within the graphitic layers and gives rise to the breaking of these layers into small pieces, as depicted in Fig. 5. Presently, Hummer’s method is regarded as the most agreeable method in comparison with the Brodie and Staudenmaier methods. Also, GO has been used in various modified forms. GO is composed of a crystalline region and a non-graphitic region owing to the oxidized groups which break the conjugated network and prevent π-electron conductivity, which makes GO insulating. Oxidized graphene is hydrophilic, contains many hydroxyl groups, and forms hydrogen or ionic bonds with polymers. The available literature on oxidized graphene-based composites indicates their broad applications in catalysis, supercapacitors, drug introduction, and the flexible film making industry.^19,20^ Moreover, graphene oxide can be used as a selective membrane through which only water molecules can pass.^21^ The magnetic behavior of GO has been observed in the limits of diamagnetism (DM) and ferromagnetism (FM) depending on the degree of graphitization and the individual nature of the functional groups. In this context, the GO preparation method plays a crucial role in controlling these functionalities, and the extent of these functionalities decides the magnetism in GO. Some examples of the magnetic interactions observed in GO are shown in Table 1.

**Table 1:** XPS ratios of sp^2^, hydroxyl (C–O), carbonyl (C=O) and carboxyl (C–O–O) groups, and the magnetic nature of GO

Material	Synthesis	(C=C) sp^2^ (area%)	(C–O) (%)	(C=O)*/(C–O–O) (%)	sp^2^/sp^3^	Magnetic nature	Moment (emu gm^−1^)	Spin density (spins per g)	Ref.
GO	Brodie method	73.7	17.3	9*	2.8	SG		—	22 and 156
GO (heated for 24 h, 180 °C)	Brodie method	86.7	10.2	3.1*	6.52	PM		—	23 and 156
NaOH treated GO	Brodie method	55.8	32	12.2*	1.26	PM		—	23 and 156
GO	Brodie or and Hummer’s method	23.32	45.33	1.48/10.94*	1.23	PM + DM	0.75	20.2 × 10^18^	22 and 157
GO	Pyrolysis of silk cocoon	—	—	—	—	(FM)	0.005	—	24
BGO	Brodie method	—	—	—	—	(AFM) + (PM)	—	2 × 10^18^	25
HGO	Hummer’s method	—	—	—	—	(AFM) + (PM)	—	2 × 10^19^	25
Monolayer GO quantum dots	Oxidative cutting	59.5	2.5	0.5*/30.5	—	PM + FM	0.159	5.7 × 10^18^	26
GO	Modified Hummer’s method	—	—	—	—	SG	—	—	27

### 2.3 Reduced graphene oxide

**Fig. 6:**
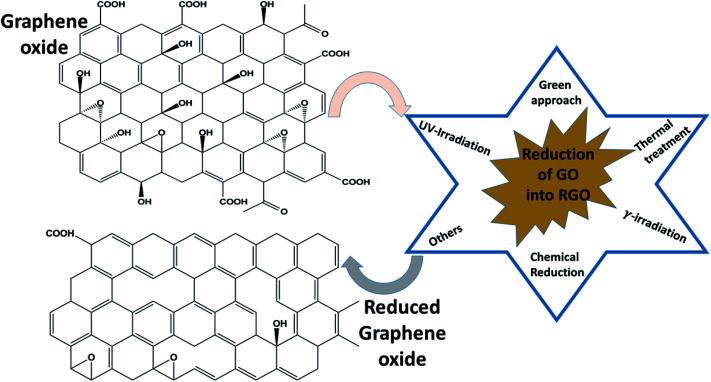
Methods of reducing graphene oxide to reduced graphene oxide.

Among all graphene derivatives, reduced graphene oxide (rGO or RGO) can be believed to be the best candidate for potential applications due to its ease of synthesis and structural similarity with graphene. Reduced graphene oxide (RGO) is the form of GO that can be obtained after the removal of oxygen functionalities from GO by applying chemical, thermal or other treatment. The physical properties of RGO strongly depend on the degree of reduction. Thus, RGO demonstrates both insulating and conducting behavior, according to the percentage of oxygen remaining on the graphene, and GO exhibits fully insulating behavior. Even though RGO has significantly lower conductivity and mobility (5000 cm^2^ V^−1^ s^−1^) than high-quality graphene obtained by mechanical exfoliation or synthesized by another high accuracy preparation method, it has a reasonably large surface area, good biocompatibility, a reliable and cost-effective synthesis, and the possibility of a large number of remaining organic groups and defects, and it offers a broad range of benefits compared with other graphene derivatives. Reduced graphene oxide is widely used in energy storage applications like supercapacitors, Li-ion batteries, solar cells, electrocatalysis, photocatalysis and many others. To date, several methods of reducing GO into RGO have been published, as shown in Fig. 6. γ-irradiation, UV irradiation, green approaches, chemical reduction and annealing have been adopted by researchers to restructure the characteristic graphitic sp^2^ network from oxidized graphene, along with some other approaches. Among all processes, the chemical reduction method is believed to be a more convenient and inexpensive way for the mass formation of RGO with good capacity, particularly compared with other reduction methods such as thermal reduction, which has minimum equipment and laboratory requirements for annealing. Apart from this, this method can even be performed at room temperature. Thus, the chemical procedure is supposed to better than the others. In the chemical reduction process, reducing agents like sodium hydroxide (NaOH), sodium borohydride (NaBH_4_), hydrazine monohydrate (NH_2_NH_2_·H_2_O), hydroxylamine (NH_2_OH), hydroquinone (C_6_H_6_O_2_), phenylenediamine (C_6_H_8_N_2_) and hydrohalic acid (HX where X = fluorine, chlorine, bromine) are used to expel the oxygen functionalities.^28,29^Table 2 shows the magnetic behavior of some RGO materials.

**Table 2:** Magnetic behavior and reduction routes of reduced graphene oxide

Material	Synthesis	Reduction method	Magnetic nature	*M*_s_ (emu g^−1^)	Ref.
RGO	Modified Hummer’s method, chemical route	NaBH_4_	(SPM)_5 K_ + (FM)_300 K_	0.06	30
RGO	Modified Hummer’s method, reflux	Hydrazine hydrate + NaOH	(FM)_300 K_	0.007	31
RGO film	Modified Hummer’s method	Ascorbic acid	(DM to FM)_10 K_	—	32
RGO	Coconut shell charcoal	700 °C	(SPM)_300 K_	0.2619	33
RGO	Modified Hummer’s method	800 °C/2 h	(PM)_10 K_ + (DM)_300 K_	—	34
GDY	Cross coupling reaction on Cu surface	—	PM	—	35

Whether induced magnetism appears in the RGO sheet depends on the fabrication process of the RGO sheet. If the reduction process of GO to RGO left defects in the graphene sheet, then the induced magnetism may be significant. However, sometimes chemical modifications are not able to affect the magnetic properties of RGO sheets due to better reconstruction of the sp^2^ bonding network. For example, Felix *et al.* reported a diamagnetic nature of RGO similar to that of pristine graphene. They synthesized the RGO by thermal reduction of GO and found a negative susceptibility of −2.17 × 10^−5^ m^3^ kg^−1^ in the as-prepared RGO.^34^ Moreover, the diamagnetism was found to decrease further on moving from 30 K to a lower temperature (∼10 K), which indicates that surface modification could not alter the magnetic properties of the as-prepared RGO. On the other hand, Sarkar *et al.* obtained super-paramagnetism (SPM) and hysteresis at 5 K and 300 K in NaBH_4_-reduced RGO.^30^ The region may contain abundant defects that were attained by the chemical reduction process. It is anticipated that clusters of defects that couple ferromagnetically behave like a single domain at low temperature, leading to the SPM. While at room temperature, other defects also effectively induce a magnetic moment, which gives rise to ferromagnetism but with a lower magnetic moment.^30^

### 2.4 Graphdiyne

**Fig. 7:**
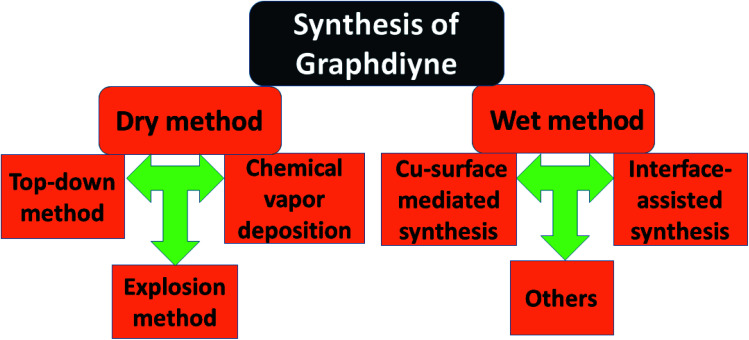
A schematic representation of the syntheses of graphdiyne based composites.

**Fig. 8:**
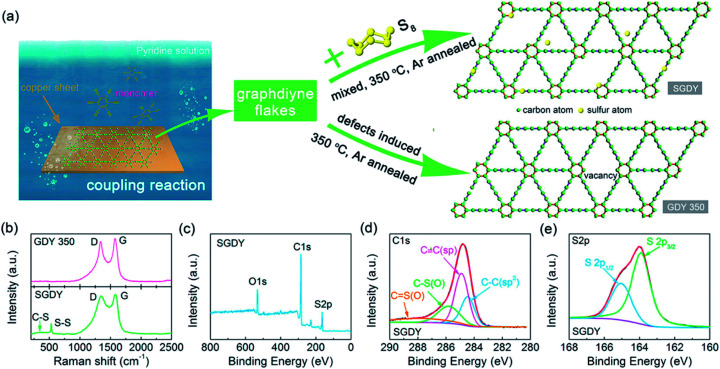
(a) Schematic depiction of the synthesis of GDY by a cross-coupling reaction on the surface of copper, (b) Raman results for GDY-350 and SGDY. (c) Wide XPS spectrum of SGDY, (d) narrow C 1s XPS spectrum of SGDY and (e) narrow S 2p XPS spectrum of SGDY. Reproduced with permission from ref. 45, copyright 2019, American Chemical Society.

Nowadays a synthetic 2D carbon allotrope, named graphdiyne (GDY), has attracted significant attention from researchers due to its planar structure.^36–39^ GDY can be made from the graphene structure just by inserting a diacetylenic linkage between two benzene rings, in contrast to the linear acetylenic chains in the graphyne structure. It is the most stable non-natural carbon allotrope containing diacetylene bonds. It consists of sp and sp^2^ hybridized carbon atoms which make it different and fascinating compared with other carbon allotropes containing sp^2^ hybridized carbon atoms such as graphene and carbon nanotubes (CNTs). In 2010, Li *et al.* first reported the successful synthesis of GDY with a direct band gap (0.46 eV), in contrast to graphene with zero band gap, and significant carrier mobility at room temperature (10^4^ to 10^5^ cm^2^ V^−1^ s^−1^). Interestingly, GDY shows stacking (AA, AB and ABC stacking) dependent physical properties in the case of multilayers.^40^ Afterwards, this motivated extensive studies on potential applications in water remediation, electronic devices, gas separation, Li-battery storage, metal free catalysis,^41,42^ sensors and solar cell devices.^43,44^ Unlike graphene, graphdiyne can be considered a promising material, particularly in spintronics. This is due to easily achievable modification in the form of substitution or doping, which provides a tunable band gap and conductivity. For instance, Chen and coworkers have shown that Cr and Mn adatoms could be easily introduced on the corner sites of GDY due to high migration barriers.^46^Fig. 7 shows some reported methods for preparing GDY. They belong to two categories: dry and wet methods. The top-down, CVD, and explosion methods correspond to the first category. On the other hand, the interface assisted and copper-surface mediated techniques correspond to the wet method category. Some other techniques have also been studied. Among them, the Cu mediated method is the most studied technique due to the ease of preparation. In this method, GDY is grown on a copper foil surface. The process takes place in pyridine through a cross-coupling reaction of the hexaethynylbenzene monomer. Fig. 8(a) shows the synthesis of sulfur doped GDY by this reaction. It has been established that pristine graphdiyne displays typical paramagnetic behavior with *M*_s_ ∼ 0.51 emu g^−1^ at low temperature (2 K), resulting from the C matrix and sp hybridization of GDY. After the annealing of pristine GDY at 600 °C, an increment in spin density leads to antiferromagnetism in GDY. The source of the magnetism in annealed GDY is recognized as the hydroxyl groups located in the chains of the GDY sheet, but the high barrier energy of 1.73 eV for OH hampers the clustering of these groups and promotes antiferromagnetism in GDY.^46^ Another research group^40^ prepared pristine GDY by annealing in hydrogen, and studied the effect of ABC stacking on its electrical and magnetic properties. They observed a direct band gap (0.64 eV) and spin-half paramagnetism at 2 K for the ABC stacking style, attributed to residual OH groups on the chains of the GDY sheet, in contrast to the previously reported direct band gap of 0.73 eV and nonmagnetic nature of GDY sheets. It is noteworthy that doping induces a localized state in carbon-related materials. As a result, paramagnetic N doped graphdiyne showed an enhanced magnetic moment almost two times greater than that of GDY.^47^ Moreover, ferromagnetism with *T*_c_ > 350 K was seen in raw Fe/GDY and annealed Fe/GDY hybrids, resulting from the presence of low valence Fe ions. Annealing of Fe/GDY increases the remanent magnetization while decreasing the coercive field with respect to the raw Fe/GDY hybrid, even at room temperature. The ferromagnetic nature of GDY based hybrids may bring forth a new way of making spin devices.

## 3 Experimental tools

The origin of magnetism in 2D carbon based composites is highly dependent on the presence of disorder as we explained earlier. Various characterization techniques play an important role in knowing the extent of defects, adatom positions, doping concentration, the nature of the dopant, *etc.*, and in determining the cause of induced magnetism in 2D carbon composites. Some important techniques are:

### 3.1 X-ray diffraction (XRD) technique

XRD is a very powerful technique that can be used for the structural characterization of 2D carbon related composites. It gives information on phases, structures, crystallinity, grain size, strain, texture and crystal disorder. Pristine graphite shows the (002) and (004) reflection peaks at 2*θ* = 26.6° and 2*θ* = 54.5° in the XRD pattern, with *d*_002_ = 3.34 and *d*_004_ = 1.68 Å, as studied by Sebayang *et al.*^48^ After the oxidation of pristine graphite, due to the oxygen containing functional groups along with water molecules, the (002) and (004) peaks shift to lower angle values of 2*θ* = 11.8° (*d*_002_ = 7.49 Å) and 2*θ* = 42.2° (*d*_004_ = 2.14 Å), respectively. After the reduction of GO to RGO by using different reducing agents as explained in an earlier section, the (002) and (004) peaks shift to higher angles of 2*θ* = 24.4° (*d*_002_ = 3.64 Å) and 2*θ* = 42.8°, respectively, due to the removal of oxygen containing functional groups. The intensity of the (002) peak plays a crucial role in providing information about the number of layers. The number of layers (*N*_L_) in GO or RGO sheets can be estimated by the following Debye–Scherrer equation:4
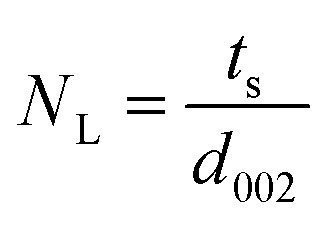
where *t*_s_ is the thickness of the graphitic stack and *d*_002_ is the interlayer spacing. *t*_s_ is given by5
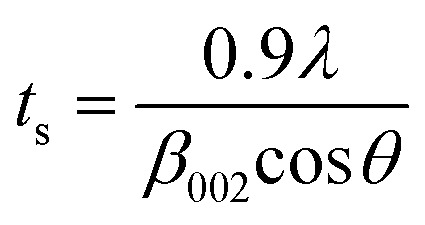
where *β*_002_ is the full width at half maximum (FWHM) that can be obtained by Lorentzian fitting of the (002) peak.^49^ This means that greater broadening of the (002) peak indicates a lower number of graphene layers. Nevertheless, a high intensity (002) peak for graphite sheets, even in graphite oxide, indicates the stacking of several sheets.

### 3.2 Microscopy techniques

Several microscopy techniques including scanning electron microscopy (SEM), transmission electron microscopy (TEM), atomic force microscopy (AFM) and scanning tunneling microscopy (STM) are used for morphology imaging of 2D carbon related materials. These are:

#### 3.2.1 Atomic force microscopy (AFM)

AFM is widely used for structural analysis and enables us to identify the number of layers in graphene sheets. Observations reveal that single-layer graphene’s thickness varies from 0.3 to 1.5 nm. Interestingly, the occurrence of functional groups in 2D materials can be discerned from the thickness variation because interactive forces between functional groups and the AFM tip in trapping mode increase the thickness. For example, the thickness of the layers in GO varies from 3 to 5 nm owing to different oxygen functionalities such as –O–, –COOH, *etc.*

#### 3.2.2 Scanning tunneling microscopy (STM)

STM gives topographical information on 2D composites with atomic resolution. Moreover, STM investigates the charge density around the Fermi level. The bias voltage between the tip and the sample decides the occupancy of states around the Fermi level. For instance, a positive bias voltage probes the lowest unoccupied states, while a negative voltage probes the highest occupied states of the specimen, depending on the tunneling of electrons either from the tip into the specimen or from the specimen to the tip.

#### 3.2.3 Scanning electron microscopy (SEM)

The exfoliation process and re-stacking lead to deformation of GO sheets, resulting in well defined crumples and ripples in the 3D interconnected network of the GO structure. On the other hand, the morphology of chemically reduced graphene sheets highly depends on the reduction method. Observations reveal that after the removal of oxygen groups, the layer structure of RGO becomes more compact, irregular and folded. This restacking and entanglement of layers is observed due to removal of the organic groups present among them. Wrinkles and curling in the graphene sheet cause it to manifest its intrinsic behavior, because blending of the 2D integument structure makes it thermodynamically stable. Thus, this unique structure of RGO confers big spaces for various applications such as battery storage, microwave absorption and many others.

#### 3.2.4 Transmission electron microscopy (TEM)

TEM imaging of RGO sheets gives better visualization of the morphology. The curved sheet like structure can be seen in TEM images of RGO sheets which have a smooth surface. Some RGO images consist of lighter and darker regions within the sheet. It is expected that the lighter regions indicate mono or few layer graphene structures while the darker regions demonstrate multi-layering of graphene. Folding and wrinkles in the graphene sheet are apparent in the images. Nevertheless, distortion occurs in GO sheets due to the oxygen functionalities, even though the presence of these groups gives a diffuse ring in the selected area electron diffraction (SAED) pattern of GO. Graphene has a crystalline nature in which the [1100] plane reflects the six membered ring. As a result, the [0001] SAED pattern of RGO has six diffraction dots due to the hexagonal symmetry of the sheet.^50^

### 3.3 Raman spectroscopy

Raman spectroscopy techniques play an important role in carbon allotropes and help us to investigate the chemical modifications including structure preservation, defects and organic groups, and also enable us to determine the undesirable synthesis byproducts. Any kind of change in the 2D carbon lattice structure leads to the Raman scattering of phonons that can be observed by variation in the intensity, shape and position of the Raman signal peaks. The Raman spectra of carbonaceous materials mainly consist of two G (∼1600 cm^−1^) and D (∼1355 cm^−1^) band signals. The G and D peaks occur due to bond stretching of the sp^2^ C–C bonds and breathing of the sp^2^ C–C bonds. The D band requires defects for its stimulation, but its second order overtone 2D (∼2635 cm^−1^) does not require defects to be active. Thus, the intensity of the 2D peak decreases with increasing number of defects in contrast to the D peak. Hence, defect-less carbon structures exhibit only the G band along with the 2D band.^51^ The 2D peak occurs at around 2700 cm^−1^. Apart from this, the combination of D and G peaks can be seen at ∼2950 cm^−1^ and its intensity depends on the defect concentration, similar to the D band peak. It has been established that shifting of the G band peak from 1600 cm^−1^ to lower wavenumber indicates the presence of p-type charge carriers in 2D graphene based composites along with recovery of the sp^2^ region in GO. Moreover, the *I*_D_/*I*_G_ ratio can be used to calculate the average size of the sp^2^ graphitic region in RGO structures using this equation:

6 *L*_D_^2^ = 1.8 × 10^−9^*λ*_L_^4^[*I*_D_/*I*_G_]^−1^

where *L*_D_ and *λ*_L_ indicate the average size of the sp^2^ domain and the wavelength of laser light, respectively. Fig. 8(b) depicts the D (1360 cm^−1^) and G (1567 cm^−1^) band peaks of GDY-350 and SGDY. In the Raman spectra of GDY-350 and SGDY, the D and G peaks result from structural defects and E_2g_ vibrational modes. Moreover, the *I*_D_/*I*_G_ ratios of both GDY-350 and SGDY are found to be larger in comparison with that of pristine graphdiyne (GDY). This enhancement results from the introduction of disorder such as vacancies within GDY-350 and SGDY.

### 3.4 X-ray photo-electron spectroscopy (XPS)

XPS is one of the most important spectroscopy techniques. The presence of adatom/substituted element or oxygen functionalities in GO and their respective oxidation state can be determined by this technique. In general, C 1s peaks are found at 280–290 eV. Peak at 284.6 eV indicates the C=C sp^2^ bonded carbon in 2D graphene sheet. After the oxidation of graphite, C 1s peaks appears ∼284/285 eV, ∼286.4 eV, ∼287.7 eV and ∼289.1 eV corresponding to sp^2^/sp^3^-carbon, C–OH, C=O, COOH organic group in graphene oxides.^52^ The ratio of the O 1s and C 1s peak intensities is used to determine the oxygen content in graphene oxide based composites. The peak intensity ratio changes after the reduction of these functionalities. As a result, the intensity of the sp^2^-C peak increases and those of the hydroxyl and epoxy group peaks decrease, whereas the intensity of the COOH peak may or not change significantly, depending on the COOH amount. After the doping of any element like nitrogen or sulfur, several sub-peaks appear in the spectrum. For instance, nitrogen doped graphene oxide exhibits three sub-peaks at ∼398.3 eV, ∼400 eV, and ∼401.4 eV which can attributed to pyridinic, pyrrolic, and quaternary nitrogen, respectively. Fig. 8(c–e) show the XPS spectra of defective GDY and S-doped GDY annealed at 350 °C. The C 1s and O 1s peaks mainly originate from adsorbed oxygen, while an S 2p peak appears at ∼164 eV, signifying the presence of sulfur atoms in GDY. Meanwhile, the high-resolution C 1s XPS spectrum of SGDY has four C–C (sp^2^), C≡C (sp), C–S (or C–O), and C=S (or C=O) peaks. On the other hand, the S 2p peak consists of two different peaks at 163.5 and 164.5 eV, ascribed to the S 2p_3/2_ and S 2p_1/2_ peaks of C–S–C. These bonding peaks represent strong bonding between C and S elements in their respective chemical states.

### 3.5 Electron paramagnetic resonance (EPR)

EPR is also known as electron spin/magnetic resonance (ESR or EMR) spectroscopy. It is a powerful method to study 2D carbon composites. EPR gives crucial information about the features of various types of paramagnetic species by estimating the number of unpaired electrons. Moreover, it is capable of investigating organic free radicals, defects and transition metal ions in 2D carbon based composites. Basically, EPR probes the interaction of magnetic dipoles with an applied magnetic field and electromagnetic radiation of the appropriate wavelength. EPR is concerned with the splitting of electronic spin states. In general, some parameters are used to characterize the EPR spectrum that not only provide information about the nature of the paramagnetic centres but also about their surroundings.^53^

#### 3.5.1 Zeeman interaction

The energy of the Zeeman interaction is described by the *g* factor which leads to splitting of the energy levels in paramagnetic materials. The energy Δ*E* (under the resonance conditions) required to reverse the direction of the electron spin in applied magnetic field *B*_0_ is equal to the energy *hν*.

7 Δ*E* = *hν* = *gμ*_B_*B*_0_

Here *h* and *μ*_B_ are the Planck constant and the Bohr magneton, respectively. On increasing the applied field *B*_0_, EPR signals can be attained. The *g* factor is inversely proportional to *B*_0_ (*i.e. g* ∝ 1/*B*_0_). For free electrons in a vacuum, the *g* value remains constant *i.e. g*_f_ = 2.002. However, *g* varies from the free electron *g* value in a paramagnetic molecule due to the impact of spin–orbit interactions. The *g* value increases considerably with increasing atomic number, and is particularly large for the lanthanides. A sharp and narrow EPR peak was observed for GO due to the localized free radicals caused by lattice defects. After the reduction of GO, a broad peak appears for RGO due to delocalization of π-electrons. The broad peak indicates the presence of induced magnetism within the RGO sheet.

### 3.6 Magnetic circular dichroism (MCD)

MCD spectroscopy is basically based on the Faraday effect due to the electromagnetic nature of light. According to Michael Faraday, the plane of polarized light in any substance will rotate in the presence of a magnetic field. Therefore, MCD demonstrates the different absorption of left and right circularly polarized light. The applied magnetic field (*H*_o_) leads to different absorption according to the direction of light propagation (parallel or anti-parallel). Chan *et al.* studied the magnetic properties of Co/graphene by using X-ray magnetic circular dichroism (XMCD). They observed moderate enhancement of the XMCD asymmetry and magnetization with Co nanoparticles in the graphene assembly, indicating dipolar-mediated magnetism.^54^ Eelbo and coworkers investigated the magnetic properties of single atoms and clusters of Fe, Co, and Ni on monolayer graphene^55^ using X-ray magnetic circular dichroism (XMCD) techniques. The Fe and Co adatoms exhibited paramagnetism and easy rotation about the out-of-plane axis. In contrast, Ni monomers showed a nonmagnetic ground state, but an increase in clustering of significant magnetic moments was observed due to intra-atomic charge transfer and hybridization effects within the adatoms/graphene.

### 3.7 Superconducting quantum interference device (SQUID)

The SQUID is a very important and sensitive magnetometer that can be used to measure extremely subtle magnetic fields and is even capable of detecting the electromagnetic energy of the human body based on superconducting loops. The ultra high sensitivity of this device relies on measuring the variation in the magnetic field allied with one flux quantum. Two parallel Josephson junctions form when two superconductors are separated by thin insulating layers, and the magnetic flux (*φ*) is quantized in these Josephson junctions according to:8
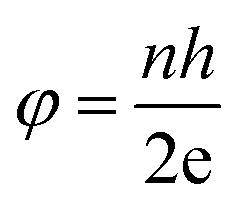


where *n* is an integer, *h* is the Planck constant and e is the magnitude of the electron charge.

### 3.8 Vibrating sample magnetometer (VSM)

**Fig. 9:**
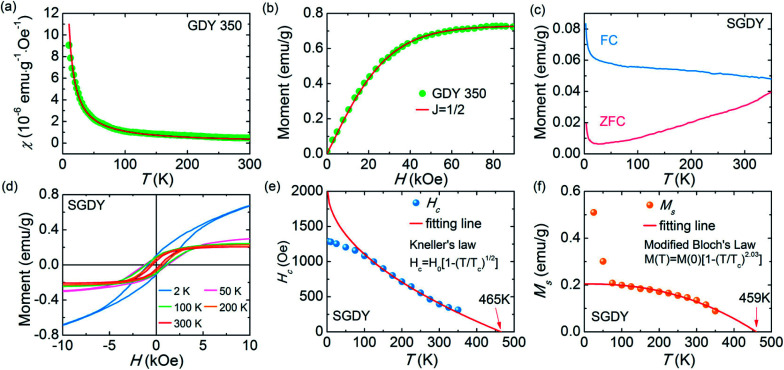
(a) Typical susceptibility–temperature (*χ*–*T*) curve for GDY-350. (b) *M*–*H* curve for GDY-350 measured at temperature 2 K. (c) Magnetization *versus* temperature (*M*–*T*) curves for SGDY. (d) Magnetization *versus* field (*M*–*H*) curves for SGDY. (e) Coercivity variation with temperature for SGDY. (f) *M*–*T* curve fitted by the modified Bloch’s law. Reproduced with permission from ref. 45, copyright 2019, American Chemical Society.

In 1955, Simon Foner invented the vibrating sample magnetometer (VSM). VSM measures the magnetic properties of carbon based materials by converting the dipole field of the sample into an AC electrical signal. When a material is placed in the uniform magnetic field, a dipole moment, which is proportional to the product of external field and susceptibility, is induced in the material. Fig. 9(a–f) show the magnetic properties of GDY-350 and sulfur-doped GDY (SGDY) measured by a PPMS-VSM (Quantum Design). Pristine GDY powder was treated with the same annealing process as SGDY, and is named as GDY-350. Fig. 9(a) shows that the magnetic susceptibility of GDY-350 decreases with increasing temperature, following the Curie law for paramagnetic characteristics. Fig. 9(b) shows the *M*–*H* curve for GDY-350 measured at 2 K that can be given by the paramagnetic Brillouin function as follows9
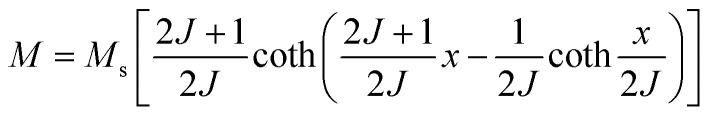


The solid line in Fig. 9(b) indicates that the experimental data are well fitted with *g* = 2 and *J* = 1/2, suggesting the contribution of defects such as vacancies or edge defects to the magnetization. In contrast, SGDY shows typical ferromagnetic behavior with *T*_c_ above 350 K (Fig. 9(c)). Further, the intrinsic paramagnetic behavior of GDY appears below 100 K. The *M*–*H* curves shown in Fig. 9(d) confirm the above results. A robust ferromagnetic ordering was obtained at room temperature with an *M*_s_ value of 0.047 emu g^−1^. For better understanding, Fig. 9(e) shows the *H*_c_(*T*) plot given by Kneller’s law, while Fig. 9(f) shows the modified Bloch law that was used to obtain *T*_c_, which was determined to be nearly 460 K, indicating robust ferromagnetic coupling in S doped GDY. It has been established that pristine graphdiyne displays typical paramagnetic behavior with *M*_s_ ∼ 0.51 emu g^−1^ at low temperature (2 K), resulting from the C matrix and sp-hybridization of GDY. After the annealing of pristine GDY at 600 °C, an increment in spin density leads to antiferromagnetism in GDY. The source of the magnetism in annealed GDY is recognized as the hydroxyl groups located on the chains of the GDY sheet, but the high barrier energy of 1.73 eV for OH hampers the clustering of these groups and promotes antiferromagnetism in GDY.^46^

## 4 Theory of magnetism

**Fig. 10:**
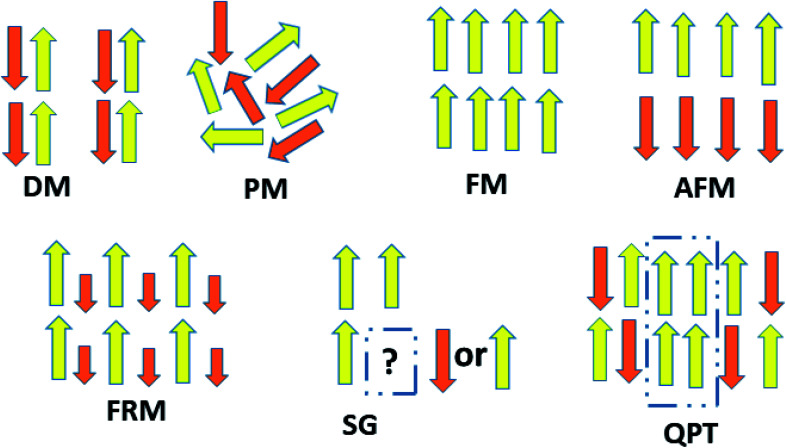
A schematic representation of magnetic interactions in 2D carbon materials.

The behavior of a material in the presence of an external field decides the typical magnetism of the material. Materials which are weakly repelled by an external magnetic field are called diamagnetic materials. This type of magnetism is known as diamagnetism (DM). These materials produce a weak magnetic field due to changes in the orbital motion of electrons. On the other hand, materials which are slightly attracted to an external magnetic field are known as paramagnetic materials, and this magnetism is known as paramagnetism (PM) (Fig. 10). Paramagnetic materials contain unpaired electrons. This leads to an interaction between the angular momentum and spin of the electron, which results in weak alignment of the magnetic moment with the applied magnetic field direction. In the case of ferromagnetism (FM), the material is strongly attracted to a magnet and is magnetized in the field direction. Moreover, ferromagnetic materials exhibit the some magnetic moment, even at zero applied field.^56^ When the magnetic moments of neighboring electrons point in the opposite direction, then this kind of magnetism is known as antiferromagnetism (AFM). Thus, antiferromagnetic materials have zero net magnetic moment. Ferrimagnetic materials possess some net magnetic moment even in the absence of an external magnetic field because atoms of these materials have opposing magnetic moments, but these opposing magnetic moments are unequal. Different theories have been proposed by scientists to explain the exact nature of magnetic materials. These fall into two categories: classical and quantum theories. Langevin explained successfully the diamagnetic as well as paramagnetic behavior of a substance, but could not explain the origin of spontaneous magnetization in ferromagnetic materials. Even Langevin’s function does not fit very well for many systems. Moreover, some other properties like magnetocrystalline anisotropy could not be explained. After this, quantum theories of diamagnetism and paramagnetism were proposed by Larmor, Van Vleck, Brillouin and Pauli. In these cases, Langevin’s function was improved, and the origin of the internal magnetic field was explained to some extent. The magnetization is given by10
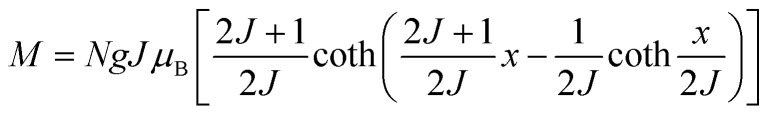
where *x* = *gJμ*_B_*H*/*kT*, and *g* is the Lande *g* factor. The function11
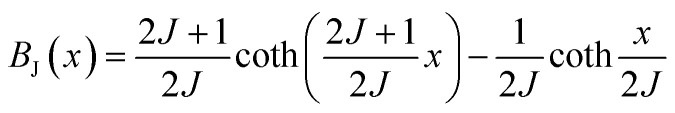
is the Brillouin function. The term *B*_J_, magnetization, can be given by:

12 *M* = *NgJμ*_B_*B*_J_(*x*)

when *x* ≪ 1, then 
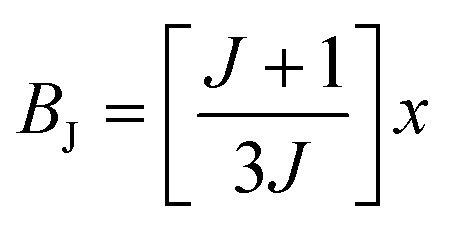
. Thus, susceptibility can be written as:13
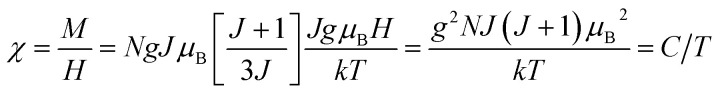
where *C* is the Curie constant *i.e.*14
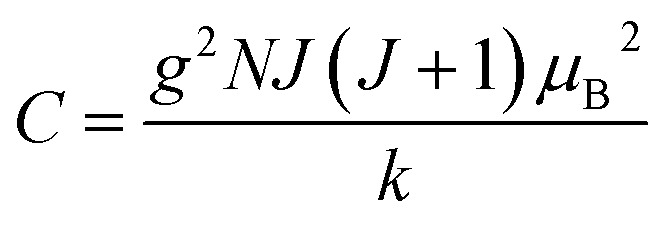


After this, in 1907 Weiss tried to explain spontaneous magnetization by molecular field theory. He introduced the concept of the internal field caused by neighboring atomic moments and added a correction in the Curie law *χ* = *C*/*T* where *C* is the Curie constant. On substituting the *H*_m_ = *γM* term in the equation *H* = *H*_tot_ − *γM*, the Curie–Weiss law for magnetic susceptibility takes the form:15
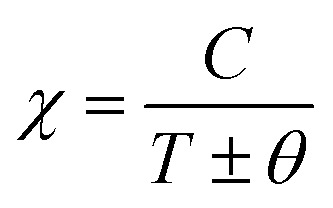
Here, *θ* = 0 defines paramagnetic susceptibility, while −*θ* indicates ferromagnetic interactions and +*θ* describes antiferromagnetic interactions as predicted by Neel in terms of the Neel temperature. These theories explained paramagnetic, ferromagnetic, antiferromagnetic, and ferrimagnetic interactions well. However, the source of the internal magnetic field could not be explored. A satisfactory explanation of the internal field was given by Heisenberg by considering exchange interactions in the model Hamiltonian according to16
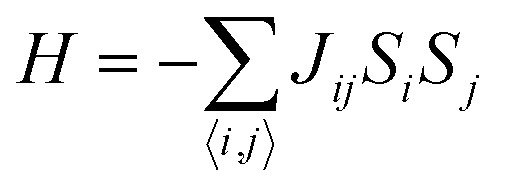
where *J*_*ij*_ is the exchange integral, and *S*_*i*_ and *S*_*j*_ are the spins of nearest neighbors. When a system is disordered and frustrated as shown in Fig. 10, then the Edwards–Anderson model gives17
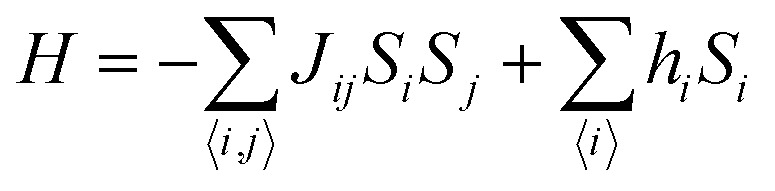
where *S*_*i*,*j*_ are classical vector spins and *J*_*ij*_ are independent random interactions taken from a characteristic distribution. Here, if *J*_*ij*_ > 0, then all spins become parallel. Then the ground state would be ferromagnetic.

If *J*_*ij*_ < 0, then the neighboring spins become anti-parallel. Then the ground state would be antiferromagnetic.

When a random mixture of antiferromagnetism and ferromagnetism occurs, then the system reaches a frustrated and disordered state which is known as a spin glass (SG) state.

Theoretically, graphene is diamagnetic. However, modified graphene shows critical magnetic behavior due to randomly oriented and unsaturated surface spins. Recently, Biswal and coworkers have shown magnetic frustration in GO prepared by the modified Hummer’s method. They have shown the coexistence of different magnetic states at different temperatures and fields. Now let us think about defect-free graphene. Since it is a pseudogap system, then a question arises as to what happens when some magnetic impurity is adsorbed on the graphene surface. This situation is different from normal metals and insulators. For metals, the magnetic moment of the impurity-atom disappears below a characteristic temperature, known as the Kondo temperature, due to screening of the local moment by the cloud of electrons. While in insulators, the moment remains unscreened at every temperature. Theorists predict the possibility of a quantum phase transition between a local moment and a Kondo-screened state as a result of coupling of conduction-band electrons. Recently, this was shown to be true by experiment. Jiang *et al.* provided evidence for Kondo-screening and a quantum phase transition between screened and unscreened phases through the vacancy magnetic moments in graphene.^57^

## 5 Computational perspective

The emergence of magnetism in 2 dimensional carbon materials can be understood by numerical and analytical approaches. In this context, the tight-binding model (*e.g.* the quasi-atomic minimal basis set orbital (QUAMBO) approach),^58^ the Hamiltonian model, the Hubbard model along with mean-field approximation (Monte Carlo, Hartree–Fock, re-normalization, *etc.*), density functional theory (DFT) methods, linearized Dirac formalism, *etc.* are some widely used methods that give information about the electronic and magnetic properties of a system. The most common density functional theory (DFT) first principles based methods use the density instead of the wavefunction. DFT is widely used to study magnetic carbon nanostructures. For this purpose, several public computer codes like the GAUSSIAN, VASP, SIESTA, WEIN2K, and CRYSTAL packages are implemented.^59–61^ Among them, the Hubbard model is known as a simple model in solid-state physics.^62^ This model was proposed by John Hubbard in 1963, and describes how interacting electrons lead to magnetic, insulating, or superconducting states in solids. The mean-field Hubbard model is known as a tool for physicists studying magnetic properties in sp^2^ carbon materials. This model is based on π-electronic symmetry states of 2D carbon. The unhybridized p_*Z*_ atomic orbitals form these electronic states in sp^2^ carbon atoms. Mathematically, the Hubbard model Hamiltonian can be represented by

18 *H* = *H*_hop_ + *H*_int_

where *H*_hop_ defines the tight-binding Hamiltonian of the nearest-neighbor.19
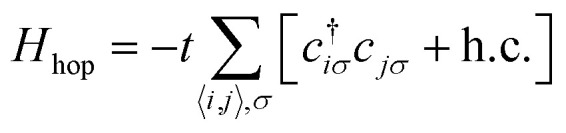
Here *c*^†^_*iσ*_ and *c*_*jσ*_ are the annihilation and creation operators with electron spin *σ* at sites *i* and *j*. The notation 〈*i*,*j*〉 stands for the pairs of nearest-neighbor atoms while h.c. is the Hermitian conjugate counterpart. The term *t* = *t*_*i*,*j*_ = *t*_*j*,*i*_ represents the quantum mechanical probability that an electron hops from site *i* to *j* (or from *j* to *i*). It is considered that hopping integral *t* ∼ 2.7 eV determines the energy scale of this Hamiltonian.

The electronic structure of sp^2^ hybridized carbon atoms can be explained by the tight-binding model. Besides, on-site Coulomb interactions contribute to the magnetism phenomenon. Therefore, the interaction Hamiltonian *H*_int_ in the mean-field Hubbard model represents a non-linear interaction that raises the energy by *U*_*i*_ when two electrons occupy a single-orbital state at *i*. The interaction Hamiltonian *H*_int_ is written as20
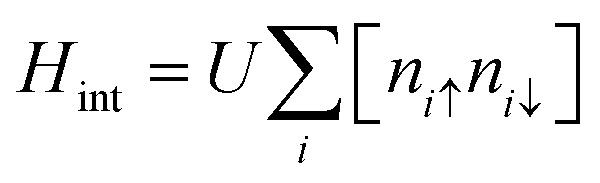
where *n*_*i*↓_ = *c*^†^_*iσ*_*c*_*jσ*_ is the spin-resolved electron density at site *i*, and the parameter *U* > 0 is a constant describing the on-site Coulomb repulsion. The main problem with this model is the lack of long-range Coulomb interactions because it considers only short-ranged nearest-neighbor interactions. To overcome this problem, the mean-field approximation has been taken into consideration. This is because the mean-field approximation allows a spin-up electron at site *i* to interact with the average spin-down populated electrons 〈*n*_*i*↓_〉 at the same site and *vice versa*. Thus, the Hamiltonian in this approximation is21
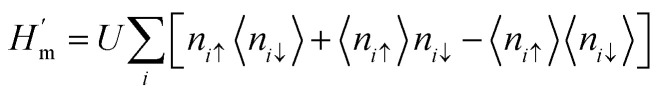


This formula effectively holds diagonal terms. This situation could be made more self-consistent by arbitrarily taking 〈*n*_*i*↑_〉/〈*n*_*i*↓_〉 values. Let us assume a situation where the selected value of 〈*n*_*iσ*_〉 disrupts the spatial symmetry of spin; it is predicted that in this case, AFM solutions would be achieved.^63^ Therefore, the approximate guess of 〈*n*_*iσ*_〉 is very important, since a suitable choice of 〈*n*_*iσ*_〉 can allow the iterations to converge very easily and pave the way for achieving a solution. The spin density at each *i*-atom is estimated self-consistently, and this is followed by obtaining22
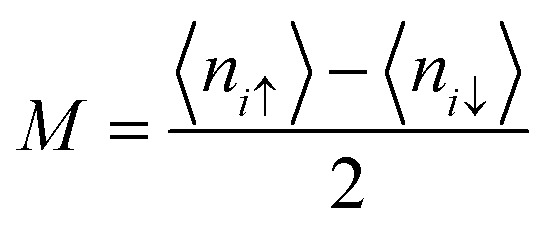
where 
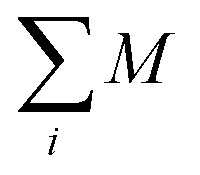
 is the total spin of the system. Since the induced magnetic moment relies on *U*/*t* values, a correct guess for *U*/*t* can give similar solutions to those obtained from other first-principles computations. The same questions on the applicability of the above mentioned models can arise when insisting on more clarifications. Does the mean-field approximation work for 2D carbonaceous materials? How can the results of a method that takes into account all electrons be compared with it? The most important question is: how to choose *U*/*t* so that converged solutions are easy to obtain. The results obtained from the mean-field approximation can be validated by comparing them with the results attained by Monte Carlo simulations or some other approximation methods. Moreover, a mindfully chosen *U*/*t* value can result in superior matching between the mean-field approximation and other *ab initio* methods such as GGA, LDA, *etc.* It is important that the consideration of all electrons in *ab initio* methods can be omitted. Nevertheless, in the case of hyperfine interactions, the equivalence of all electrons plays a crucial role because 1s carbon atoms contribute effectively to spin polarization. For instance, a chosen *U*/*t* of ∼1.3 gives similar results to the generalized gradient approximation (GGA) method of the DFT model, while *U*/*t* of ∼0.9 is found to give similar results to those computed by the local spin density approximation of the DFT model.^61^ It should be noted that *U*/*t* ≥ 2.23 gives a Mott–Hubbard transition to AFM ordering in the honeycomb lattice.^64^ Apart from this, an investigation of the tight-binding Hamiltonian of the honeycomb lattice can be carried out using benzenoid graph (BG) theory, which can be assumed as an alternative to the mean-field Hamiltonian. The BG theory is basically based on counting principles. The graph’s nullity is given by the following equation:

23 *η* = 2*α* − *N*

Here *N* and *α* are the total and the possible number of sites. In the tight-binding model, the number of zero-energy states can be predicted by the above formulation. Furthermore, the Stoner criterion is used to determine the evolution of the magnetism. The Stoner criterion tells us about the losses and gains of kinetic energy and exchange energy in a system due to spin polarization. For a given value of the exchange splitting *A*, one can estimate the moment by employing the self-consistency condition *i.e.*24
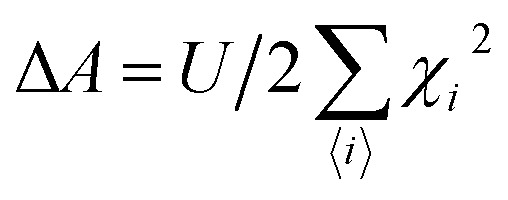
where 
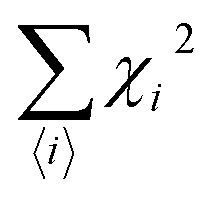
 measures the degree of localization of the corresponding state. Wang *et al.* observed large spin in arbitrarily shaped graphene nanoflakes as a result of topological frustration of π-bonds. They used the benzenoid graph theory and first-principles calculations for computation. However, the benzenoid graph theory has some limitations *e.g.* it is not capable of aligning the spins in particular states.^65^ In 2D carbon materials, the emergence of magnetism is governed by Lieb’s theorem. Apart from the benzenoid graph theory, Lieb’s theorem can give the total spin of the carbon based system. Interestingly, Lieb’s theorem is valid in all dimensions and does not require periodicity of the crystal lattice. According to this theorem, if the bipartite system is half-filled, then its ground state can be characterized by total spin *S* = 1/2[*N*_A_ − *N*_B_], where *N*_A_ and *N*_B_ stand for the numbers of sites in sublattices A and B.^66^ In general, whether we use the DFT or the Hubbard model, the Coulomb interaction term plays a crucial role in determining the magnetism. Wehling *et al.* have demonstrated the effect of the local Coulomb interaction strength *U* on the chemical bonding and magnetic moments of transition metal (TM) adatoms on graphene. They used the GGA and GGA+U methods for computation. For Fe, Co, and Ni adatoms on graphene, the electronic configuration is determined by the Coulomb potential *U*. It is established that 4s electrons are fully de-localized for 3d adatom decoration on normal metal surfaces and become part of the conduction electron sea. The situation is quite different for 3d adatoms on graphene. In this case, half-filled 4s orbitals are part of the impurity spin. Thus, co-exixstence of high-spin (HS) and low-spin (LS) solutions for Fe, Co, and Ni results in different ground-state configurations due to the frustrated 4s orbitals of the adatoms on graphene.^67^ On the other hand, He and coworkers have shown the impact of *U* on 3d TM adatoms on graphdiyne (GDY) by the DFT+U method. The modulation in the *U*_eff_ = (*U* − *J*) value from 5.23 to 6.63 for Z/GDY where Z = V, Cr, Mn, Fe, Ni, Co shows the variation in electronic properties between metallic, semiconductor and semi-metallic states. The magnetic moments are in the order Cr (4.85 *μ*_T_) > Mn (3.79 *μ*_T_) > V (3.34 *μ*_T_) > Fe (2.46 *μ*_T_) > Co (1.00 *μ*_T_) > Ni (0.0 *μ*_T_). The strong coupling between the TM and GDY leads to electron re-arrangement between the TM orbitals and electron injection from the TM to GDY which gives rise to the overall magnetic moments.^68^

## 6 Source of magnetism in 2D carbon

To fabricate flexible and durable information devices, graphene and its derivatives are believed to be excellent materials for next-generation spin-based devices. This is because the excellent carrier mobility, and weak spin–orbit and hyperfine coupling lead to long coherence time and diffusion length in 2D carbon materials. The major hindrance is the lack of intrinsic magnetism which limits the spin relaxation length. All carbon materials have diamagnetic susceptibility leading to intrinsically nonmagnetic behavior due to delocalization of π-band electrons. This means that breaking of these delocalized π electronic systems can effectively engineer magnetism in carbonaceous materials. Thus, creating sp^3^-type defects such as point defects (vacancies/adatoms), multiple defects (cracks, voids or zig-zag edge states), and topological defects including pentagons, heptagons or both, or creating terminal groups at the edges could result in localized magnetic moments in 2D carbon-related materials. Additionally, negative or positive Gaussian curvature and surface ripples or corrugation also affect the magnetism. For instance, the covalent functionalization of GO creates an sp^3^-network with unsaturated dangling bonds that act as a source of magnetism in GO. Hence, magnetism in graphene-based materials with no d or f electrons can be tuned in different ways, as discussed in the later subsections.

### 6.1 Role of defects

Before the discovery of graphene, it was believed that structural defects could cause instability for dimension d less than or equal to 2 due to long-wavelength fluctuations, as stated by the Mermin–Wagner–Berezinskii theorem. However, 2D graphene is found to be a stable structure and does not follow the theorem. Like its 3D counterparts, structural defects in 2D graphene and its derivatives can dramatically change their optical, electrical, and magnetic properties. The defects may be intrinsic or extrinsic. In the 3D crystal system, when the crystalline order is disturbed without the presence of foreign atoms, then it is referred to as an intrinsic defect. The presence of foreign atoms in the 3D lattice is referred to as an extrinsic defect. Hence, graphene follows whatever is valid for the 3D crystal. Therefore, both intrinsic and extrinsic defects have contributed to the overall 2D magnetism in graphene and its derivatives. Defects are associated with unsaturated dangling bonds that have great impact on the physical properties of graphene derivatives due to the scattering of electron waves at the defects. The defects may be in the form of:

#### 6.1.1 Stone–Wales defects

**Fig. 11:**
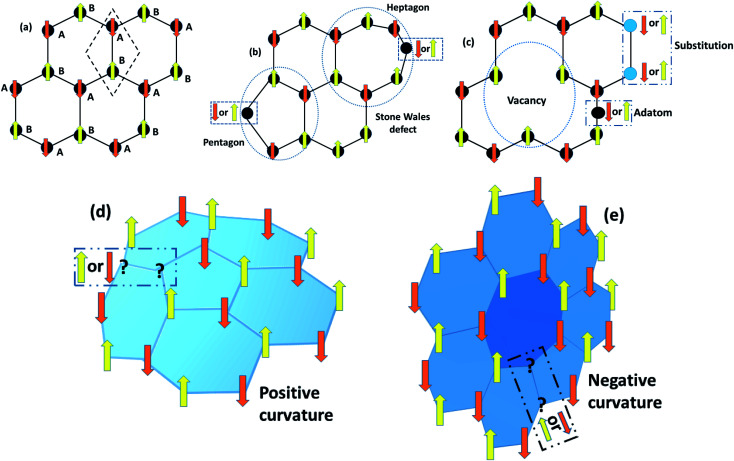
Schematic diagrams of Lieb’s theorem for a 2D bipartite lattice. (a) The rhombus unit cell of graphene, shown by a dashed box, has two A and B sub-lattice points that localize with opposite spins for energy minimization. These opposite spins result in antiferromagnetic interactions with the same number of A and B sub-lattice points. Nevertheless, the bipartite characteristics collapse in the case of (b) Stone–Wales defects, (c) vacancies, adatoms and substitution defects in graphene, (d) positive Gaussian curvature due to pentagons, and (e) negative Gaussian curvature resulting from heptagons, which lead to unusual magnetic properties.

The graphene lattice is known to have the capacity for reconstruction by forming non-hexagonal rings without adding or removing an atom. For example, 90° rotation of the C–C bonds transforms four hexagons into pentagon and heptagon pairs.^69^ The above structural irregularities create an imbalance due to the lack of spin pairs, which gives rise to the localized density of states, as depicted in Fig. 11(b). Among all defects, Stone–Wales defects are predicted to be more favorable owing to the low formation energy of around 5 eV.

#### 6.1.2 Topological defects

Pristine graphene has zero Gaussian curvature owing to the perfect lattice arrangement of the rhombus unit cell. In a bipartite system, the unit cell consists of A and B sub-lattice points that localize with opposite spins to attain a stable structure. The same number of A and B sub-lattice points causes the antiferromagnetic ground state that gives a net magnetic moment equal to zero, as shown in Fig. 11(a). However, the occurrence of five and seven membered rings instead of six membered rings disturbs the bipartite characteristics as a result of defects. In this case, the system will either go to the high-spin state or the low-spin state, and we cannot define the exact nature of the sub-lattice which is responsible for the critical magnetic properties in a bipartite system like 2D graphene. In unballasted graphene, non-hexagonal rings induce local Gaussian curvature in a graphene sheet. For example, pentagons induce positive curvature, while heptagons lead to negative curvature (Fig. 11(d and e)). This Gaussian curvature leads to the unpaired spins being either spin up or spin down, and they can act as localized moments within graphene and its derivatives. When these unpaired spins interact with other adatom or defect spins, then complicated magnetic behavior that depends on the type of magnetic interaction can be seen in 2D carbon materials.

#### 6.1.3 Vacancies

The introduction of vacancies is the simplest and most fundamental method to induce magnetism. A simple missing atom is known as a lattice vacancy or a Schottky defect. Double vacancies can be achieved by removing two neighboring atoms or by the coalescence of two vacancies. They may be extended or multiplied with the occurrence of more missing atoms.

#### 6.1.4 Adatoms

Frenkel defects or transfer from one lattice site to another interstitial position do not occur in a single layer of graphene. High energy would probably be required for transferring an atom to an in-plane interstitial position. For instance, moving a C atom to the hexagon center requires more energy in comparison to the bridge position. A carbon adatom appears instead of an interstitial defect (Fig. 11(c)).

#### 6.1.5 Substitution

The introduction of foreign atoms into the graphene sheet is well known to result in substitution impurities. Nitrogen doping can be considered as an example of this type of defect.

#### 6.1.6 Line defects

When graphene is grown by CVD, irregular growth results in different crystallographic orientations in several positions. This is known as line defects in graphene.

**Fig. 12:**
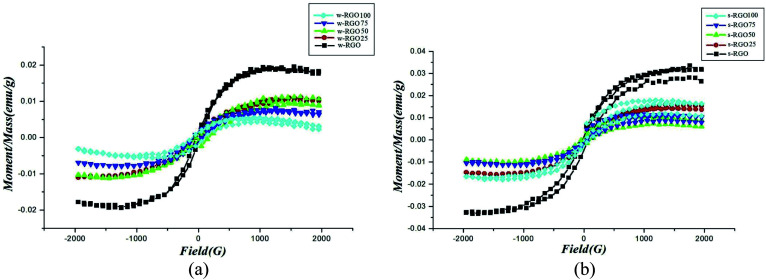
Magnetization *versus* field measurements for (a) w-RGO and (b) s-RGO at different γ-irradiation doses. Reproduced with permission from ref. 70, copyright 2019, Elsevier.

This kind of defect may be introduced into graphene in several ways, *e.g.*, by electron irradiation, γ-irradiation, proton irradiation, N-ion irradiation, thermal annealing or rapid cooling in high-temperature environments, chemical treatments, *etc.* The defects are very useful up to a limit. Nevertheless, high concentrations of defects may adversely affect the lattice arrangement of 2D carbon. Defects may alter the bond lengths of the inter-atomic valence bonds and damage the stability. This can further limit the magnetic properties of the material. Fig. 12(a and b) show the *M*–*H* curves for w-RGO and s-RGO at different γ irradiation doses of 25, 50, 75, and 100 kGy. The γ rays were generated by ^60^Co with a gamma-quanta energy of 1.23 MeV. Here, w-GO (weak) and s-GO (strong) indicate the oxidation of graphite in the presence of graphite : KMnO_4_ ratios of 2 : 3 and 2 : 8, respectively, while the reduced GO is represented by w-RGO and s-RGO, respectively. It is clear from Fig. 12(a) that w-RGO exhibits superparamagnetic behavior before and even after γ-irradiation. As the gamma dose increases, the magnetization starts decreasing steeply after the initial irradiation and at a slower rate for higher doses. The magnetization is expected to increase because the level of structural defects is enhanced by irradiation. This contrary result shows that the magnetic properties not only depend on structural defects but also rely on other factors like oxygen functionalities. Therefore, hydroxyls (OH) and epoxides (–O–) could have a larger effect on the superparamagnetic magnetization (SPM) in the irradiated samples in comparison to structural defects. At higher γ doses, the number of structural defects is reduced significantly due to the restoration of sp^2^ carbon networks. Further, the decrease in SPM for 75 kGy irradiated samples may be due to the lower density of defects having a greater influence on the SPM with respect to the higher oxygen content. At higher irradiation dose (100 kGy), the disruption of carbon bonds creates a new sort of structural defect. This is because the oxygen functionalities get a chance to escape from the graphene lattice, which restores the lattice to its perfect arrangement. As a result, oxygen is released at 100 kGy doses, which causes a decrease in SPM. Fig. 12(b) shows the ferromagnetic features of irradiated s-RGO. After 25 kGy to 50 kGy doses, the ferromagnetism drastically decreases due to the reduction in the number of defects, and the density of OH/–O– groups remains almost unchanged in s-RGO-25. After a 50 kGy irradiation dose, a large number of OH/–O– groups may be removed from the graphene, giving rise to deoxygenated defects. Hence, s-RGO-50 contains lower oxygen content but a higher amount of deoxygenated defects than s-RGO-25. In comparison with s-RGO-50 and s-RGO-75, irradiated sample s-RGO-100 shows enhanced ferromagnetic character. An increase in the level of defects and a higher amount of hydroxyl groups as compared to the previous dose may cause more ordering.

### 6.2 Chemical modification by hydrogenation or halogenation

Hydrogenation or halogenation is known to be the simplest way of chemical modification of graphene based derivatives. The similar electronegativities of C and H (hydrogen) do not disrupt the electronic structure of graphene significantly after doping. However, fully hydrogenated graphene is predicted to be diamagnetic like pristine graphene, and semi-hydrogenated graphene was shown to be ferromagnetic. Thus, controlled doping of hydrogen atoms in 2D carbon materials may produce magnetic moments by ferromagnetic coupling that induces a long-distance ferromagnetic ordering in H-doped graphene materials.^71–73^ Similarly, absorption of fluorine can induce a magnetic moment, depending on the concentration of fluorine adatoms, as explained in a later subsection.^74^

#### 6.2.1 Hydrogenation process

Hydrogenation of 2D carbon based materials is known to be an effective way to induce a magnetic moment with the advantage of reversibility.^71^ The fully hydrogenated derivative of graphene, designated as graphane with (C_1_H_1_)_n_ composition, has been studied by several authors and exhibits attractive properties.^75^ It would be predicted that semi or fully hydrogenated graphene sheets exhibit tunable ferromagnetic and band gap properties. Like zigzag graphene, semi or partially hydrogenated graphene contains unsaturated π bonds that induce ferromagnetism. Two strategies, phase hydrogenation of graphene and liquid phase hydrogenation or exfoliation of graphite, are widely used to prepare partially hydrogenated graphene. The first process results from the hydrogenation of graphene below 10% using H_2_ at different pressure and temperature.^76,77^ Xie *et al.* investigated the magnetism in partially hydrogenated graphene. Interestingly, the benefit of the partial hydrogenation is the formation of unpaired electrons in graphene, which induces magnetism within graphene and its derivatives. These unpaired electrons along with other remnant delocalized bonds improve the ferromagnetic properties of 2D graphene derivatives, as observed in partially hydrogenated epitaxial graphene by Xie *et al.*^76^ On the other hand, Eng *et al.* produced highly hydrogenated graphene (C_1.38_H_1_O_0.28_) through the Birch reduction of graphite oxides.^77^ This kind of hydrogenated graphene demonstrates weak ferromagnetism along with an antiferromagnetic contribution. Eng *et al.* have shown that magnetism occurs due to the hydrogenation of graphene, not because of any metallic impurities in hydrogenated graphene.

#### 6.2.2 Fluorinated graphene

Similar to hydrogenated graphene, fluorographene (CF)_*n*_ is versatile in diverse applications in energy conversion and prevents graphene derivatization owing to chemisorption of fluorine (F) atoms.^78^ The magnetic and electric properties of fluorinated graphene rely on the concentration and distribution of F adatoms.^79^ It corresponds to either a p-type metal (with/without the ferromagnetic spin arrangement) or a large-gap semiconductor (without magnetism), depending on the concentration and distribution of adatoms. Feng and coworkers experimentally observed a high magnetization of 0.83 emu g^−1^, a high magnetic moment of 3.187 × 10^−3^*μ*_B_ per carbon atom and a high efficiency of 8.68 × 10^−3^*μ*_B_ per F atom in fluorinated reduced graphene oxide. This may be attributed to the many vacancies, which hinder the clustering of F atoms and introduce many magnetic edge adatoms.^74,80,81^ For fluorinated graphene quantum dots (GQDs), obtained by thermal cutting of fluorinated graphene, strong paramagnetism occurred because of sp^3^-type defects and magnetic zigzag edges in the GQDs. It was anticipated that F adatoms in GQDs are most likely incased at the edges which have high efficiency for bringing in paramagnetic centers in the GQDs-F system.^82^

#### 6.2.3 Halogenation of 2D graphene

As well as fluorine, another way of functionalizing graphene is halogenation by the other halogen elements like chlorine, bromine, and iodine.^73,83^ F-doped graphene can be obtained by the chemical reaction method by heating a mixture of XeF_2_ and 2D graphene sheets. It has been reported that fully fluorinated graphene can be synthesized with a C : F ratio of 1 : 1, but for the other halogen elements, partially halogenated graphene is experimentally obtained. The covalently bonded halogens significantly modify the intrinsic properties of graphene, such as electrical conductivity, mechanical strength, optical transparency, carrier mobility, and chemical stability. Overall, halogenation is a very versatile method to tailor graphene for its intended application. The interaction between the halogen molecule and the graphene is of vital importance in the effective halogenation of graphene. Although the synthesis routes might be different for various halogenated graphenes, the halogen source is usually an atmosphere containing the halogen diatomic molecule. Therefore, the strength and nature of the interactions between halogen molecules and graphene have aroused intensive interest from a computational point of view. Rudenko *et al.* have investigated the adsorption of fluorine, chlorine, bromine, and iodine diatomic molecules on graphene using density functional theory calculations. This study reveals that van der Waals correction plays a crucial role in the estimation of the binding strength of the halogen molecule. The in-plane orientation of the molecules has been found to be more stable than the orientation perpendicular to the graphene layer. Nguyen and coworkers determined the effect of halogen adsorption on graphene nanoribbons. The results revealed that the magnetic/non-magnetic states such as ferromagnetic or antiferromagnetic metals are highly influenced by the concentration of adsorbed halogen (X: Cl, Br, I, At) atoms and the edge structure.^84^ Initially, on increasing the adatom concentration, the number of holes per unit cell increases. Thereafter, beyond 60% adsorption, the magnetic state is transformed into a nonmagnetic state. It was predicted that adatoms, the carbon edge, and (X–X)/(X–C) bonds induce spin states within the halogen/graphene nanoribbon and are responsible for tuning the physical properties of halogen/graphene nanoribbons.

### 6.3 Role of the organic group in magnetism

**Fig. 13:**
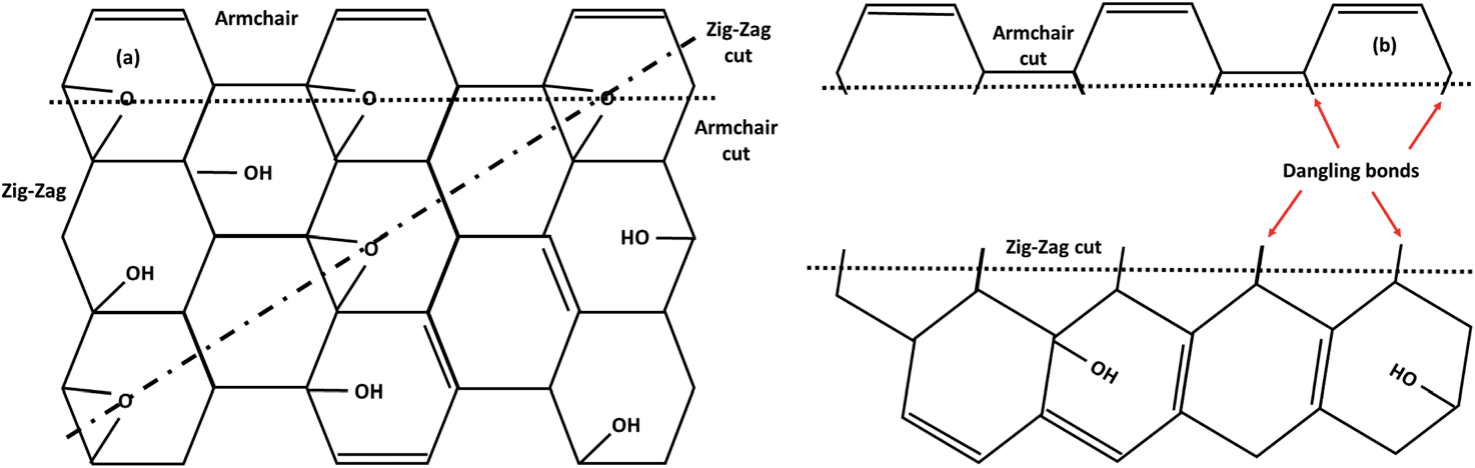
(a) A pictorial representation of unzipping of graphene oxide *via* (b) zig-zag and/or armchair cutting.

**Fig. 14:**
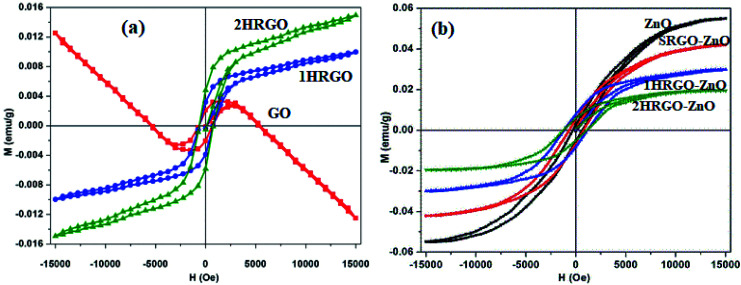
*M*–*H* curves of (a) GO, 1HRGO and 2HRGO, and (b) ZnO, SRGO–ZnO, 1HRGO–ZnO and 2HRGO–ZnO nanocomposites, reproduced with permission from ref. 90, copyright 2018, Springer.

Organic groups such as oxygen-containing hydroxyl (–OH), carbonyl (C=O), carboxyl (–COOH) and epoxy (C–O–C) groups are known to be an excellent source of magnetism in graphene-related materials. During the oxidation of graphite, these functionalities go into the graphene skeleton. It is anticipated that carbonyl and carboxyl groups occur at the periphery of the GO sheet, while the interior region of the graphene is populated by hydroxyl groups and epoxy groups (in the basal plane). Theoretical investigations revealed that a major contribution to the ferromagnetism in GO comes from hydroxyl groups and hydroxyl clusters.^85^ Graphene is a bipartite system; the distribution of the hydroxyl groups randomly occurs on the carbon atoms. This random arrangement of OH groups breaks the A, B lattice symmetry, giving rise to a localized state with uncompensated spin. Hence, a single OH can produce a magnetic moment. If we think about an epoxy group in which the oxygen bonds with an adjacent carbon atom at the edge, it preserves its symmetry and does not contribute to the total moment. The generation of hydroxyl groups in sufficient amounts for creating a large number of uncompensated spins on the basal plane of the graphene sheet is hard to achieve in practice. In this direction, researchers have tried to bring epoxy groups into a line; when the epoxy group bonds with a C–C bond along a line, then the C–C bond breaks and forms a C–O–C ether structure. After this, the reduced GO sheet unzips to form either zigzag or armchair edges,^86^ as depicted in Fig. 13(a and b). Two research groups, Pan *et al.* and Bagani and coworkers, unzipped graphene/GO sheets by a hydrothermal method and a thermal annealing process, respectively. The cutting/unzipping was found to be responsible for increasing the number of zigzag edges.^87^ Zigzag edges are important like defects because, at the edges, the electrons are energetically degenerate with highly localized states and unpaired spins giving rise to local moments at the edge boundaries to minimize the Coulomb energy. Another way of increasing the number of OH groups on the basal plane is the ring opening of the epoxy group, which has been proposed by Shin *et el.*^88^ They effectively generated a high content of hydroxyl groups by using sodium borohydride (NaBH_4_) as an alkaline reagent with GO. On the other hand, Chen *et al.*^89^ introduced 10.74% hydroxyl groups and observed superior enhancement of the density of localized spins from 0.4 to 5.17 *μ*_B_/1000 C. It could be said that tuning of oxygen-containing groups within 2D carbon materials may be an effective way to obtain superior magnetic properties. The major hindrance of attaching a large number of groups is the clustering of these groups resulting from the low barrier energy acquired from the pristine graphene. Further, this depletes the magnetic moment of graphene and makes the electrical properties more complicated. Fig. 14(a) shows the *M*–*H* plots of hydrothermally reduced RGO obtained after eight hours at 180 °C. Here, 160 mg of dispersed GO (1 mg/1 mL) and 320 mg of dispersed GO (2 mg/1 mL) products are designated as 1HRGO and 2HRGO, respectively. GO has weak ferromagnetism with an *M*_s_ of 0.003 emu g^−1^ and a major diamagnetic contribution. Usually, GO consists of unreacted sp^2^ regions along with reacted sp^3^ regions. Partial distortion of layers results from oxygen groups like epoxy, hydroxyl, carbonyl, and carboxyl in the reacted region, and is responsible for the magnetic moment. It was anticipated that a weak magnetic moment arises as a result of interacting magnetic regions and domains. Magnetic regions consist of magnetically active edge states that act as localized spin and vacancies with unsaturated dangling bonds, while energetically favorable epoxy groups on the opposite side of the hexagonal ring and OH groups attached in the basal plane of GO contribute to the overall moment. Nevertheless, carbonyl and randomly distributed hydroxyl groups at the edge sites and on the basal plane of GO effectively lead to a diamagnetic contribution. So far, as we know, the removal of organic groups enhances the edge state and creates defects in RGO in the form of vacancies, Stone–Wales defects, or structural distortion. At the same time, the number of π–π stacking sites increases as a result of the restoration of sp^2^ carbon conjugations. Overall, the high density of defects contributes to the total spin moment in RGO sheets. Thus, 1HRGO showed strong ferromagnetism with *M*_s_ ∼ 9.05 memu g^−1^, while 2HRGO had a high *M*_s_ of ∼15.05 memu g^−1^. Such a high moment in 2HRGO comes from the magnetic anisotropy energy density and long-range magnetic interactions that might be due to the tiny crystallite size and the large number of grain boundaries among small portions of the RGO sheets.^90^ On the other hand, a first principles study on hydroxylated graphdiyne reveals that the magnetic properties of GDY survive only at low –OH concentration, since hydrogen bond formation takes place at higher concentrations due to the pairing to two –OH groups. Antiferromagnetic coupling occurs between two –OH groups, which diminishes the magnetism in GDY.^91^ Sun and coworkers observed critical magnetic behavior in hydroxyl (–OH), carboxyl (–COOH), amino (–NH_2_) and thiol (–SH) functionalized graphene. For –SH, –OH and –COOH groups, the functionalized graphene was found to be in coexisting diamagnetic, paramagnetic and ferromagnetic phases at temperature 5 K. It was predicted that the complex behavior of functionalized graphene results from vacancies, edge states, chemical doping and the attached functional groups.^92^

### 6.4 Effect of multilayers on the magnetism

As we explained earlier, interfacial charge transfer between a semiconductor and RGO sheets gives rise to spin-polarized electron transport. Many semiconductors like RGO–ZnO, RGO–SnO_2_, and RGO–WO_3_ that exhibit ferromagnetic behavior have been reported so far. But the question arises as to how different ratios of carbon and semiconductor tune the magnetic moment. Fig. 14(b) shows the *M*–*H* curves of ZnO, SRGO–ZnO (95 °C for five h in DMF), 1HRGO–ZnO, and 2HRGO–ZnO (1HRGO and 2HRGO samples in DMF) nanocomposites obtained with different ratios. In comparison to SRGO–ZnO, 1HRGO–ZnO, and 2HRGO–ZnO, ZnO possesses the highest magnetic moment. This high magnetic moment in ZnO comes from oxygen/zinc vacancies and zinc/oxygen interstitial defects. However, RGO wrapped ZnO forms magnetically inactive defects owing to the non-uniform distribution of RGO sheets. After an increase in the number of RGO shell layers on the ZnO core, the ferromagnetism is further reduced. This was ascribed to the fact that decoration of RGO sheets on the ZnO surface lowers the oxygen vacancy concentration. As a result, the interparticle interactions of ZnO nanoparticles also decrease due to the wrapping of RGO layers over the ZnO nanoparticles. This causes a lack of long-range magnetic interactions within ZnO/RGO, which is responsible for the decrement in the magnetic moment (Fig. 14(b)).

### 6.5 Temperature-tuned ferromagnetism

The annealing process plays a crucial role in the ferromagnetism of graphene-based materials. Thermal energy creates vacancies and topological defects like a pentagon with uncompensated spin. The total spin *S* estimation of a defect is determined by sub-lattice imbalance, which decides the nature of the magnetism like ferromagnetism or antiferromagnetism. Nevertheless, defects of *S* = 0 spin also exhibit some local magnetic moments.^93^ The presence of structural defects breaks the symmetry of the graphene p orbital, which induces a band gap in graphene.^94,95^ Park *et al.* annealed graphene at 800 °C under an argon atmosphere. The annealed graphene showed ferromagnetic behavior with a Curie temperature of 220 K in comparison to pristine graphene which was paramagnetic.^96^ Zhao *et al.*^71^ explored the tunable ferromagnetism of hydrogenated multilayer graphene after high-temperature treatment. They predicted that during thermal treatment, the number of hydrogen atoms chemisorbed on two different sub-lattices changes, which gives rise to the magnetic moment.

### 6.6 Strain induced magnetism

Mechanical deformations significantly affect the electronic structure of graphene. In graphene, the density of states vanishes at the Dirac point, but the inhomogeneous strain can break the sub-lattice symmetry and give rise to local DOS far from the Dirac point. Therefore, it is anticipated that tuning of the mechanical force can induce magnetism in graphene-related materials because symmetry breaking induces uncompensated spin and magnetic interactions. An enhanced Kondo effect was seen in strained graphene.^97^ In comparison to Co films, Co/graphene shows a larger perpendicular magnetic anisotropy when compressive and tensile strain is applied. Yang *et al.* revealed that the tensile strain effect arises from an increase in the hybridization between the same spin d_*xy*_ and d_*x*^2^–*y*^2^_ states of the surface Co film.^98^ In the case of multi-layer armchair graphene nanoribbons, strain in the armchair edge direction induces a transition from a ferromagnetic to a nonmagnetic state when the strain reaches a critical value. Moreover, strain causes a change in bond length which decreases the stability of the edge-quantum well. Increasing the strain gives rise to spin splitting of the energy bands around the Fermi level, which decreases the spin moment.^99^ On the other hand, a semiconductor to semimetal transition in graphdiyne was discovered by Cui *et al.* through inducing strain.^100^ On applying uniaxial tensile strain, the band gap in graphdiyne decreases from 0.47 eV to nearly zero, while it increases from 0.47 eV to 1.39 eV as the result of increasing biaxial tensile strain. It was anticipated that a change in uniaxial strain leads to the breaking of geometrical symmetry which lifts the degeneracy of the energy bands. Thus, Dirac cone-like electronic structures are observed.

### 6.7 Substitutional, decoration and intercalation impurities

The incorporation of other atoms into graphene results in substitutional impurities. These impurity atoms either supersede some of the carbon atoms or take a stable place among the carbon atoms. Theorists revealed that ionic bonding occurs between alkali metal (lithium, sodium, potassium) adatoms and graphene, which has an adverse effect on the electronic states of the graphene. On the other hand, group-III metal adatoms showed mixed covalent bonding and ionic bonding which influenced the lattice of graphene a little bit. Nevertheless, 3d transition metals exhibit strong covalent bonding with graphene layers. As a result of strong hybridization between the d_*x*^2^–*y*^2^_ and d_*yz*_ orbitals of the 3d transition metal atoms and the p_*z*_ orbital of the carbon atoms, charge transfer takes place from the d-metal to the graphene substrate similar to what was observed by Liu *et al.* who showed that 0.59, 1.12, 0.73, and 0.86 electrons transfer from V, Fe, Co, and Ni adatoms to graphene, respectively. The influence of these impurities in 2D carbon is discussed in later subsections.

#### 6.7.1 Alkali metal intercalation

The alkali metals consist of lithium (Li), sodium (Na), potassium (K), *etc.* The adsorption of alkali metal atoms on 2D carbon materials has been widely studied due to the possibility of manufacturing superconducting electronic devices.^101^ For instance, Li intercalation in few-layer graphene exhibits superconductivity at transition temperature *T*_c_ = 7.4 K, while Ca-doped graphene becomes a superconductor at temperature 6 K.^102^ On the other hand, potassium doped few-layer graphene exhibits persistent superconductivity with a transition temperature of 4.5 K.^103^ Let us think about the magnetism in alkali metal–graphene. Pantha *et al.* calculated the DOS for graphene and sodium (Na) intercalated graphene sheets. They employed van der Waals interactions *via* London dispersion effects in the DFTD2 approach and used the Rappe–Rabe–Kaxiras–Joannopoulos (RRKJ) model of ultrasoft pseudopotential to account for the interaction between the ion cores and the valence electrons and the generalized gradient approximation (GGA) formalism to treat the electronic exchange and correlation effects, as described by Perdew–Burke–Ernzerhof (PBE). They found non-magnetic behavior and zero band gaps for the DOS in graphene, consistent with previous reports. For the sodium-adsorbed graphene system in a 4 × 4 graphene supercell, the DOS were not symmetrical, and the conduction band and valence band were overlapping, which indicates that the system is magnetic with a magnetic moment of 0.24 *μ*_B_.^104^ This is because some charge transfers take place from the electron donor sodium atoms to the carbon atoms of graphene.

#### 6.7.2 p-Block element doping

First, we will discuss doping with light elements like boron, nitrogen and sulfur. These elements act as intrinsic dopants in the carbon structure because there is less variation between their atomic radii and that of carbon.^47,105^ Thus, they can easily replace carbon atoms and the Fermi level is shifted according to the type of impurity, which further changes the electronic structure of graphene. Several studies have been performed on N-doped graphene sheets, graphene nanoribbons, graphdiyne, graphene oxides, *etc.* Let us consider the example of N-doped graphene in which there are three major N bonds: graphitic, pyridinic and pyrrolic configurations. Among them, graphitic and pyridinic bonding greatly influence the electronic properties of N-doped graphene, while pyrrolic N bonding introduces five-membered heterocyclic rings along with sp^3^ hybridization which enhances the electron concentration within graphene. Thus, the magnetic properties of N-doped graphene are fascinating due to the number of distinct N bonding configurations.^106^ Li and coworkers synthesized N-doped graphene with a dominant pyrrolic N bonding configuration at a N doping concentration of up to 6.02%. The pyrrolic dominant N-doped graphene showed strong ferromagnetism. It is anticipated that a large amount of doping defects may be generated in N-doped graphene, due to the presence of five-membered rings in place of six-membered rings in the pristine 2D graphene. Additionally, pyrrolic N atoms provide lone pair electrons and generate localized spin states. All the above factors improve the magnetic properties of N-doped graphene. Zhang and co-workers introduced nitrogen (5.29% N/C ratio) and sulfur (6.52%) inside graphdiyne, and this doping not only improved the saturation magnetization but also increased the long-range magnetic ordering, as summarized in Table 3.

**Table 3:** Light element induced magnetism in 2D carbon materials

Material	Synthesis	Magnetic nature	*M*_s_ (emu g^−1^)	Ref.
N doped graphene	Self-propagating high-temperature synthesis (SHS)	FM	0.148	107
N functionalized GO	Hummer’s method, microwave	FM	5.3 × 10^−3^	108
S doped graphene	Thermal treatment of GO in H_2_S	PM + FM	5.5	109
N doped GO	Chemical exfoliation, annealing at 500 °C/3 h in ammonia	FM	1.66	105
N doped GDY	Cross coupling reaction on Cu surface	PM	0.96	47
S doped GDY	Cross coupling reaction on Cu surface	FM	0.047	45
Pyrrolic N-doped graphene	Modified Hummer’s method, hydrothermal	FM	1.4 × 10^−2^	106

#### 6.7.3 Decoration with d-block elements

In addition to p-element doping, transition metal impurities such as Ni and Co, which have a much bigger radius, have also gained significant attention due to their ability to introduce charge into the p_*z*_ based electron arrangement of graphene or any of its derivatives. In general, this is a consequence of different bond lengths between carbon and other atoms, which are longer compared to carbon–carbon bonds. It was proposed by theorists a few years back that when transition metal atoms are decorated on the 2D carbon surface, a charge carrier cloud associated with the s orbital of the transition metal (TM) is partially relocated to its d orbital, and the remains are relocated to the graphene p orbital. This charge relocation is accountable for creating a potential barrier at the d-metal/graphene interface and gives rise to an antiferromagnetic interaction to minimize the magnetostatic energy. Table 4 shows the magnetic behavior of some d-metal decorated carbon substrates.^58,110–113^ Mondal and coworkers showed anti-ferromagnetic interactions in Ni/graphene and Co/graphene (Neel temperature *T*_N_ ∼ 32 K) surfaces for the first time. On the other hand, Hota *et al.*^114^ attained ferromagnetism by the charge transfer effect at the Co/graphene interface by means of porphyrin capping on the Co atoms. This was due to the compensation of the d-orbital spin of the Co atom by capping with a porphyrin molecule.

**Table 4:** Transition metal and alloy decorated graphene derivatives

Material	Synthesis	Magnetic nature	*M*_s_ (emu g^−1^)	Ref.
Fe/GDY	Cross coupling reaction on Cu surface	FM + AFM	—	115
Ni/RGO	Modified Hummer’s method, reflux method	FM	20.4	31
Pt–Ni/RGO	Modified Hummer’s method, reflux method	SPM	—	31
Pt/RGO	Modified Hummer’s method, reflux method	DM	—	31
Ni/functionalized graphene	Modified Hummer’s method, chemical reduction	FM	1.5	112
Co/functionalized graphene	Modified Hummer’s method, chemical reduction	FM	2	112
Co/graphene	Modified Hummer’s method, hydrothermal treatment	AFM	—	116
Ni/graphene	Modified Hummer’s method, hydrothermal treatment	AFM + FM	∼47	113
Co capped with porphyrin/graphene	Modified Hummer’s method, chemical reduction	FM	—	114
Graphene/Co (partially oxidized)	Heat treatment, hydrogen reduction	FM	9.3	117
Ge/graphene quantum dot	Modified Hummer’s method, microwave irradiation	PM	—	118

#### 6.7.4 Metal oxide doping

**Fig. 15:**
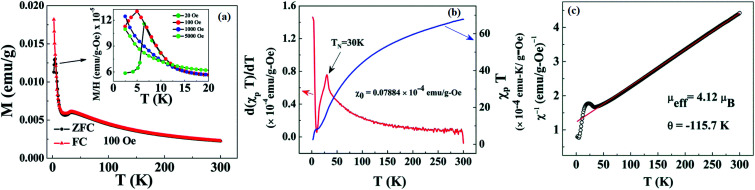
(a) Field cooling (FC) and zero field cooling (ZFC) plots for Co_3_O_4_@RGO composites obtained at field 100 Oe. The inset plot indicates the low temperature regime investigated at 20 Oe, 100 Oe, 1000 Oe and 5000 Oe. (b) The *χ*_p_*T versus T* and ∂(*χ*_p_*T*)/∂*T versus T* plot: *χ*_p_ is given by *χ* − *χ*_0_ (where *χ*_0_ = 7.08 × 10^−6^ emu g^−1^ Oe^−1^), and (c) *χ*^−1^*versus T* plot for the Co_3_O_4_@RGO composite. Reproduced with permission from ref. 120, copyright 2019, Royal Society of Chemistry.

For spin-based devices (*i.e.*, in spintronics applications), doping of metallic impurities like Co and Ni in 2D graphene cannot be considered appropriate because charge transfer from the s orbital of the d-metal to its 3d orbital makes it nonmagnetic. On the contrary, metal oxides do not have an s orbital and this prevents charge transfer. Further, the presence of oxygen assists in the formation of ionic bonding, especially in oxide ferrites. Table 5 summarizes the semiconductor decorated 2D carbon-based materials. More importantly, many of the oxides show antiferromagnetism and it is very interesting to see the effect of antiferromagnetic metal oxide decoration on a diamagnetic substrate, *e.g.*, graphene. In this context, Bhattacharya and coworkers have grown an ultrathin Ni(OH)_2_ layer on a graphene surface and observed an antiferromagnetic transition.^119^ Sarkar *et al.* found a spin-glass phase in Co_3_O_4_/RGO composites, as shown in Fig. 15(a–c).^120^ Like the FC plot, the ZFC plot shows a peak at 30 K, which is a feature of antiferromagnetic interactions. In AFM, the transition temperature is given by the Neel temperature (*T*_N_) which is characterized by a peak in the ∂*χ*/∂*T versus T* plot. Apart from the *T*_N_ peak, at low temperature the FC and ZFC magnetization plots show another peak at around 5 K (*T*_sg_, inset of Fig. 15(a)), indicating a ferromagnetic component. It was estimated that this FM component may be due to uncompensated spin at the surface of the AFM core of Co_3_O_4_ nanoparticles anchored in the RGO sheet. In comparison with 100 Oe, the ZFC magnetization measured at 20 Oe showed a peak at a somewhat higher temperature, while no peak appeared down to 2 K for applied magnetic fields of 5 kOe and 1 kOe. This kind of dependency of the ZFC peak position on the magnetic field indicates the existence of a metastable state. At *T* ≫ *T*_N_, fitting parameters were obtained using the modified Curie–Weiss law, *i.e. m*_eff_ = ∼4 *μ*_B_, *θ* = ∼116 K and *χ*_0_ = 7.1 × 10^−6^ emu g^−1^ Oe^−1^. Sarkar and coworkers have predicted that only Co^2+^ ions contribute to *m*_eff_, not Co^3+^ ions due to the lack of permanent magnetic moment as a result of 3d level splitting in the octahedral crystal field along with the pairing of 3d^6^ electrons in the t_2g_ levels. A more negative *θ* indicates antiferromagnetic exchange interactions between Co^2+^ ions. Let us think about nonmagnetic metal oxide impurities, such as SnO_2_ and WO_3_. Observations reveal that non-magnetic metal oxide nanostructures seem to be very sensitive to surface defects, which induce ferromagnetism, depending upon the surface modification.

**Table 5:** Semiconductor oxide decorated graphene derivatives

Material	Synthesis	Magnetic nature	*M*_s_ (emu g^−1^)	Ref.
ZnO/RGO	Reflux method	FM	∼ 0.01	22
G-WO_3_	Modified Hummer’s method, hydrothermal	Coexistence of FM–AFM	14.9 × 10^−3^	137
Fe_3_O_4_/RGO	Modified Hummer’s method, reflux	SPM	48.9	138
RGO/SnO_2_	Modified Hummer’s method, hydrothermal	SPM	4.6 × 10^−3^	139
Co_3_O_4_/RGO	Modified Hummer’s method, chemical route	Spin glass		120
Co_3_O_4_/RGO	Modified Hummer’s, hydrothermal method	FM + SPM	—	140
Fe_2_O_3_/RGO	Modified Hummer’s, microwave solvothermal	AFM + PM	—	130
Ni–Co-hydoxide/RGO	Chemical route	SPM	—	141
RGO/Ni–NiFe_2_O_4_	Solvothermal	SG	—	142
CoFe_2_O_4_/graphene	Modified Hummer’s method, hydrothermal	FM	∼42	143
MnO_2_/graphene	CVD	FM	1 × 10^−3^	144
CoFe_2_O_4_/graphene	One step sonication	FM	18	145
SRGO/ZnO	Hydrothermal, solvothermal	FM	40.6 × 10^−3^	90
CoO/RGO	Modified Hummer’s method, solvothermal	FM	∼7	146
Ni(OH)_2_/RGO	Modified Hummer’s method, hydrothermal	AFM–FEM	—	119
RGO/MnFe_2_O_4_	Modified Hummer’s method, hydrothermal	FM	40.5	147
RGO–ZnFe_2_O_4_	Modified Hummer’s method, hydrothermal	FM	34.5	147
RGO/Fe_3_O_4_	Modified Hummer’s method, solvothermal	FM	13.2	148
N-doped RGO–ε-Fe_3_N	Modified Hummer’s method, polyol method	SP + FM	46	149
CoFe_2_O_4_@RGO@TiO_2_	Modified Hummer’s method, hydrothermal, vapor thermal methods	FM	2.95	150
Fe_3_O_4_@TiO_2_/RGO	Chemical method	SPM	4.98	151

### 6.8 Size and shape tuned magnetic properties

**Fig. 16:**
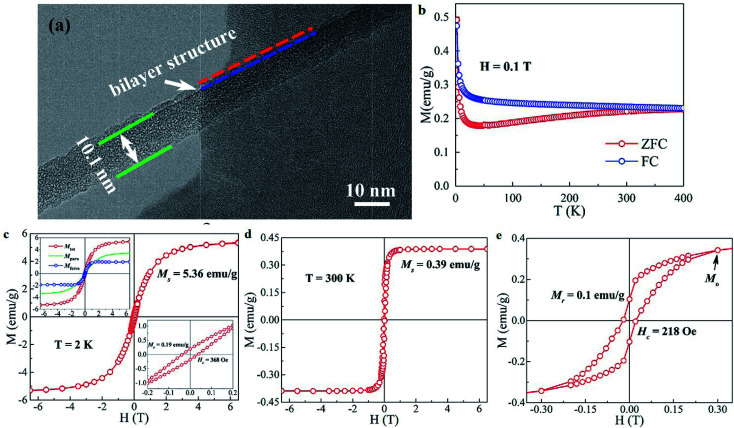
(a) TEM picture of the isolated aGONRs-400; blue and red colored dashed lines demonstrate the bilayer structure of the aGONRs-400. (b) Temperature dependent magnetization curves obtained at 0.1 Tesla field in the range 2 to 400 K. (c and d) The *M*–*H* curves obtained at 2 K and 300 K, respectively. The top left inset of (c) shows the *M*–*H* curves for the paramagnetic (*M*_para_) and ferromagnetic (*M*_ferro_) components of the total magnetization (*M*_total_). On the other hand, the bottom right inset of (c) depicts a magnified view of the *M*–*H* curve at 2 K from −0.2 to 0.2 T. (e) A magnified view of the *M*–*H* curve from −0.35 to 0.35 T at 300 K where the black arrow indicates *M*_0_. Reproduced with permission from ref. 129, copyright 2019, American Chemical Society.

Size and shape have always been crucial for tuning magnetic properties as predicted by theoretical calculations.^121^ For instance, cutting of a graphene sheet into two parts results in the formation of zigzag or armchair edges which alters the electronic properties of the sheet. The zigzag and armchair edges of graphene are seemingly like the *trans*- and *cis*-polyacetylene structures, respectively. Graphene occurs in different forms like quantum dots, graphene nanoribbons (GNRs) and graphene nanoflakes (GNFs).^122^ Graphene quantum dots (GQDs) are 0D carbon nanoallotropes within the size limit of 2 to 20 nm. The quantum confinement and edge effects are an engaging feature of GQDs. Graphene nanoribbons (GNRs) are 1D carbon nanoallotropes which have a finite width of not more than 50 nm. The role of finite-size effects and edges in determining the physical properties can be easily understood in GNRs. On the other hand, arbitrarily fragmented graphene shapes are known as graphene nanoflakes (GNFs). In general, GNFs have finite dimensions. GNFs possess several regular and irregular shapes including nanodisks, nanoplatelets and nanoislands within the 1–50 nm size range. As the size is reduced, the magnetic anisotropic effect becomes dominant. For example, tiny graphene quantum dots show superior properties in comparison with nanosheets due to quantum confinement and edge effects.^123^ Sun and coworkers observed Curie-like paramagnetism at *T* = 2 K with a local moment of 1.2 *μ*_B_. The residual zigzag edges passivated by –OH groups could be considered as the source of the magnetic moment in graphene quantum dots.^123^ GQDs may be hexagonal, triangular, circular, and randomly shaped.^124,125^ Since magnetic properties can be characterized by the electron edge states, using the tight-binding approximation, Ortega *et al.* observed two types of edge states in GQDs with different geometrical shapes, *i.e.* zero-energy (ZE) states and dispersed edge (DE) states. The ZE states are placed at the zero-energy Dirac point, while the DE states are located close to a Dirac point at not quite zero energy. GQDs with randomly shaped, hexagonal and circular structures have DE states, hence they are diamagnetic in nature. In contrast, triangular GQDs have ZE states which leads to spin paramagnetism in small dots at low temperatures. However, large size triangular GQDs exhibit diamagnetism at high temperatures.^124^ The two triangular shaped graphene dot systems embedded in an h-BN sheet indicate that GQDs prefer the closest interdot spacing as their ground state.^125^ In the GQDs-h-BN arrangement, a ferrimagnetic type of spin polarization with *S* = 1/2 occurs on each GQD and is aligned in singlet and triplet arrangements between two GQDs at interdot spacings of 5.0 Å or more. It is noteworthy that GQD and Fe_3_O_4_ nanoparticle based composites exhibited room temperature ferromagnetism. It was predicted by Sajjad and coworkers that the spin polarized edges of the GQDs and the magnetic Fe_3_O_4_ nanoparticles induced ferromagnetism in Fe_3_O_4_–GQDs composites.^126^ Similarly, zigzag graphene nanoflakes (GNFs) showed strong edge magnetism. On increasing the system size, the zigzag edge states in the GNFs become more localized and stronger.^127^ Antiferromagnetic behavior was seen in bilayer graphene nanoribbons with Neel temperature *T*_N_ = 66 K, and the spin-polarized edge states are a possible magnetic source. Further, it was anticipated that interlayer couplings give rise to antiferromagnetism in bilayer graphene nanoribbons.^128^ Recently, Fu *et al.* have seen stable ferromagnetism at room temperature by low-temperature annealing of GO nanoribbons (GONRs).^129^Fig. 16(a) shows the TEM image of GONRs annealed at 400 °C for 1 hour in the presence of argon (named as aGONRs-400). The width and thickness of aGONRs-400 were found to be ∼10.1 and 0.77–0.98 nm, respectively, which shows that a bilayer structure was obtained by annealing the GONRs. The divergence of the FC and ZFC curves reveals the ordered nature of the magnetism, while the large magnetization far from zero even at 400 K indicates a *T*_c_ high above room temperature, as depicted in Fig. 16(b). At 2 K, saturation magnetization *M*_s_ ∼ 5.36 emu g^−1^, coercive field *H*_c_ ∼ 368 Oe and remnant magnetization *M*_r_ ∼ 0.19 emu g^−1^ were obtained, clear evidence for ferromagnetic interactions within aGONRs-400 at 2 K rather than both PM and FM coexisting (Fig. 16(c)). Total magnetization can be given by *M*_total_ = *M*_para_ (3.39 emu g^−1^) + *M*_ferro_ (1.97 emu g^−1^), as shown in the inset plot in Fig. 16(c). This indicates that the ferromagnetic component is larger than the paramagnetic component. On the other hand, a pure ferromagnetic feature was seen at 300 K with *T*_c_ above room temperature, as displayed in Fig. 16(d). The high *M*_s_ of ∼0.39 emu g^−1^, *H*_c_ of ∼218 Oe and *M*_r_ of ∼0.1 emu g^−1^ strongly suggest typical ferromagnetic behavior at this temperature (Fig. 16(e)). Pure ferromagnetic behavior was observed for aGONRs-400 which has the highest *M*_s_ value of all graphene materials, which shows that the morphology of graphene-based materials has a great influence in tuning the magnetism.

### 6.9 Solvent tuned magnetic properties

The solvent may also tune the magnetic properties of graphene materials. Jedrzejewska *et al.* studied the influence of different solvents^130^ on the magnetic properties of graphene. It was seen that ethanol made graphene exhibit positive susceptibility, which means paramagnetic behavior. On the other hand, acetone made graphene show negative susceptibility, indicating the diamagnetic nature of the magnetism. From here, it was concluded that in ethanol graphene contains some magnetic moments of unknown origin. Moreover, Xu *et al.* found that carrying out the synthesis under pressure and using 1-methyl-2-pyrrolidinone as a solvent increases the production of bigger graphene nanoribbon (GNR) skeletons with nitrogen doped zigzag edges. This improves the overall magnetization within N-doped GNRs.^131^

### 6.10 Concentration dependent magnetic properties

The concentration of anchoring magnetic materials in graphene-related materials also matters, and can have a great effect on the magnetic properties. The weak magnetic interactions between the magnetic centers can be compensated by their high concentration. It has been observed that small magnetic nanoparticles with size below 10 nm act as superparamagnets with high magnetic moment due to single domain occurrence below a critical size. When the concentration of magnetic nanoparticles increases in the 2D substrate, then there is a possibility that these domains, which are distributed in various directions, may couple in such a way that might increase/reduce the net magnetic moment. On the other hand, agglomeration also reduces the magnetism.

### 6.11 Effect of heavy elements on 2D carbon magnetism

Few researchers have focused on f-block element adsorption on a graphene sheet. It is proposed that the band gap *E*_g_ can be tuned through the intercalation of heavy atoms in 2D carbon materials, which plays an important role in graphene-based nanoelectronics. It is well known that f-shell heavy elements possess silent magnetism. Thus, the magnetic properties also greatly influence the electrical properties. Sung *et al.* have shown the effect of Eu intercalation on graphene formed on a SiC (0001) substrate. The band gap arises from the hybridization between the graphene π-bands and the Eu 4f bands. It was seen at 60 K that the band gap increases due to increasing hybridization, and this hybridization occurs due to the enhanced magnetic ordering at low temperatures. Magnetic ordering can result in antiferromagnetic (AFM) and ferromagnetic (FM) structures. Both produce similar band dispersions. Nevertheless, the AFM structure was found to be better at opening a band gap in comparison to the FM structure.^132^ Wei *et al.* observed a strong interfacial exchange field in a graphene/EuS heterostructure as a consequence of strong Zeeman splitting.^133^

### 6.12 Magnetism induced by the proximity effect

**Fig. 17:**
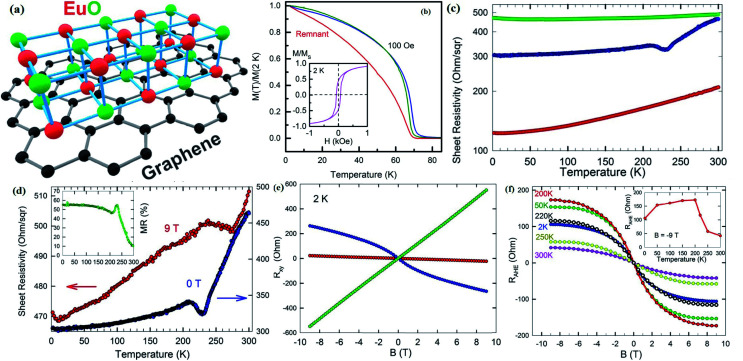
(a) Structure of EuO/graphene. (b) Normalized *M*–*T* plots measured at 100 Oe for the EuO/graphene structure and the EuO/graphene (+Eu) structure indicated in blue and green, respectively, while the inset depicts the normalized *M*–*H* plot obtained at low temperature (2 K) for EuO/graphene (+Eu) film. (c) Sheet resistivity *versus* temperature plots for pristine graphene, the EuO/graphene structure and the EuO/graphene (+Eu) structure, shown by the green, blue and red curves, respectively. (d) Sheet resistivity of the EuO/graphene structure measured at applied magnetic fields of zero and 9 T, shown by the blue and red colors, respectively, while the inset shows the magneto-resistance (MR). (e) Transverse resistance dependency on magnetic field for pristine graphene, the EuO/graphene structure and the EuO/graphene (+Eu) structure, shown by the green, blue and red colors, respectively. (f) Magnetic field and anomalous Hall effect (AHE) resistance in the EuO/graphene structure observed at *T* = 2, 50, 200, 220, 250 and 300 K, represented by the blue, green, red, black, yellow and magenta curves, respectively. The inset plot indicates the *T* dependence of the AHE resistance (at −9 T, saturated state) in the EuO/graphene structure, reproduced with permission from ref. 136, copyright 2018, American Chemical Society.

Light-weight 2D carbon-based materials are supposed to have potential applications in spintronic devices due to their high Fermi velocity (10^6^ m s^−1^), long spin coherence length of up to 10^4^ ps and poor spin–orbit coupling (SOC), which make them superior candidates for low power consumption spintronic devices. Unlike scattering from impurities, the decoration of magnetic materials on 2D carbon materials can be assumed to be a milder method to produce magnetism since the chemical bonding in graphene can be maintained. But, the magnetic materials can push current away from the carbon materials. In this regard, magnetic insulators can be used with graphene to control the motion and modulation of spin without affecting the graphene properties. Poor magnetic properties are improved by proximity interaction in 2D carbons by the interaction of magnetic insulators like europium chalcogenides (EuO), yttrium iron garnet (YIG), *etc.*^133–135^ Recently, Averyanov *et al.*^136^ have shown experimental evidence of high-temperature magnetism in graphene induced by proximity to EuO, a salient magnetic semiconductor. Fig. 17 shows the magnetic proximity spin–orbit interactions at the EuO/graphene interface. At this interface, squares of EuO couple with hexagons of graphene (Fig. 17(a)). Fig. 17(b) shows the normalized *M*–*T* plots for EuO/graphene and EuO/graphene (+Eu). Similar magnetization curves can be seen with *T*_c_ ∼ 69 K, which is close to the bulk value. Fig. 17(c) shows the sheet resistivity plots. Unlike pristine graphene, both EuO/graphene and EuO/graphene (+Eu) have lower resistivity owing to the doping effect. EuO/graphene (+Eu) showed metallic behavior, which was expected due to strong doping with Eu intercalated in graphene. Nonetheless, EuO/graphene showed a transition at *T*_c_ ∼ 220 K. Above *T*_c_ ∼ 220 K, the steeper increase in resistivity is consistent with scattering at paramagnetic centers owing to magnetic disorder. Fig. 17(d) presents the temperature dependence of the resistivity in the presence of a magnetic field. It is visible that a high field shifts the transition temperature to a higher temperature, which confirms the magnetic nature of the transition. The transverse resistance demonstrates linear behavior for pristine graphene and the EuO/graphene (+Eu) structure. This reveals that the graphene is strongly n-doped in EuO/graphene (+Eu) because of Eu intercalation, unlike pristine graphene, which is p-type. On the other hand, the EuO/graphene structure is found to exhibit nonlinear behavior. The higher carrier mobility in the EuO/graphene structure is additional evidence of transport through graphene states with both Eu and Eu oxide (Fig. 17(e)). Fig. 17(f) presents the anomalous Hall effect (AHE) resistance, whose conformation to nonlinearity is strongly reduced above *T*_c_. The above results confirm that EuO induces magnetism in graphene at a temperature that is three times as high as the Curie temperature of EuO, as a result of the proximity effect. This opens up new challenges for theorists. Meanwhile, Zanolli *et al.* investigated the proximity effect in graphene/BaMnO_3_ composites. They found a strong mutual magnetic interaction at the interface of graphene/BaMnO_3_ which induces spin polarization in graphene. This interaction also affects the magnetic substrate, down to several layers below the surface. Furthermore, BaMnO_3_ hybridizes with graphene, which causes overall magnetic softening of the Mn spin interaction. This hybridization alters the surface layers from AFM to FM, and also affects the BaMnO_3_ easy plane.

### 6.13 Gate voltage (*V*_g_)-tunable magnetism

**Fig. 18:**
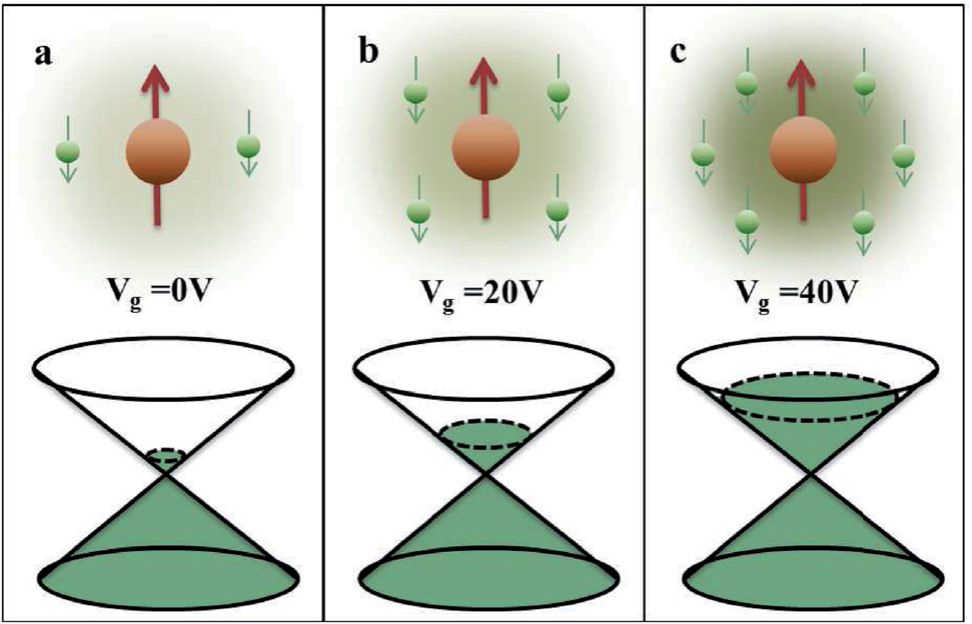
Density (
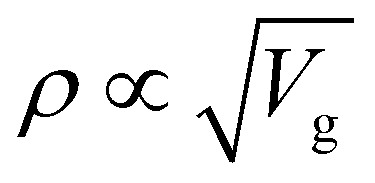
 where *ρ* is the density of graphene) dependent screening of the magnetic moment in graphene decorated with magnetic cobalt(ii) phthalocyanine molecules: (a) is at a small carrier concentration with gate voltage (*V*_g_** **) = 0 V. (b) is at a moderate concentration with *V*_g_** **=  20 V. (c) is at a large concentration with *V*_g_ = 40 V. The magnetic moment screening become stronger with increasing the carrier concentration with gate voltage, reproduced with permission from ref. 155, copyright 2018, Elsevier.

**Fig. 19:**
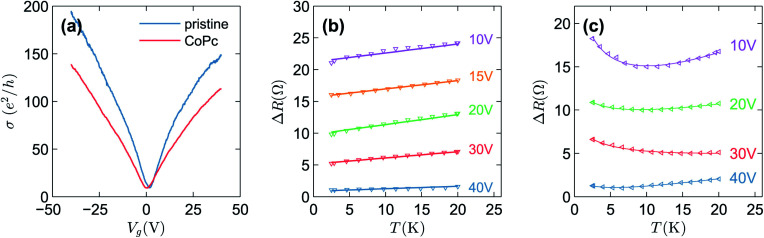
(a) Conductivity *versus* gate voltage plots for pristine graphene/h-BN (blue line) and CoPc decorated graphene/h-BN (red line). Temperature dependence of resistivity at different gate voltages for (b) pristine graphene/h-BN, and (c) CoPc decorated graphene/h-BN, reproduced with permission from ref. 155, copyright 2018, Elsevier.

The onset of magnetization is expected when the spin degeneracy of the impurity states is removed as a result of Stoner instability. Thus, the gate voltage helps to adjust the *E*_F_ to fulfill the Stoner criterion (*IN*(*E*_f_) > 1, where *N*(*E*_f_) are the DOS at *E*_F_ in the nonmagnetic case, and *I* is the Stoner parameter).^73^ Experiments show that the gate voltage effectively influences the magnetism in 2D carbon materials. It was seen in N-doped GO, α-RuCl_3_/graphene heterostructures, and graphene grafted with Pt-porphyrins, revealing the existence of ferromagnetic phases with significant magnetic moments for particular values of *V*_g_.^152–154^ However, the low-temperature competition between the Kondo effect and the indirect exchange interaction has been observed in many magnetic material doped graphene-based devices, in which the Kondo effect tends to suppress the magnetic moment while the RKKY interaction tends to maintain the spin ordering between the magnetic impurity and the substrate. In this regard, *V*_g_ helps to maintain the magnetic ordering in the system. Yan *et al.* observed a Kondo effect resulting from magnetic impurities in graphene decorated with magnetic cobalt(ii) phthalocyanine molecules as a consequence of gate voltage.^155^Fig. 18(a–c) shows how the different concentration with gate voltage affect the screening the electron in Cobalt(ii) phthalocyanine magnetic molecule decorated graphene. At low carrier concentration, strong magnetic spin flipping occurred due to poor screening of the electron, but as *V*_g_ and the concentration increase, the spin-flip scattering decreases. At sufficiently high concentration, the spin-flip scattering is indeed suppressed at temperatures below 20 K. Conductivity *versus* gate voltage plots for pristine graphene/h-BN and CoPc decorated graphene/h-BN are shown in Fig. 19. After decoration with CoPc, the Dirac point shifts slightly left by ∼1.5 V. Fig. 19(b and c) present the temperature dependence of the resistivity at different gate voltages for pristine graphene/h-BN and CoPc decorated graphene/h-BN, respectively. It is clear that an upturn in resistance emerges at low temperatures for the different gate voltages, confirming the screening of the magnetic moment at low temperature.

## 7 Conclusions

Two dimensional (2D) carbonaceous materials such as graphene and its derivatives have enormous potential in major fields of scientific research due to their intriguing properties. One of the interesting applications of 2D materials is in the field of magnetism. Although various studies are available in the literature, some parts of the above field have still not been fully exploited. This review may be helpful to provide a versatile and practicable strategy for studying of magnetic properties of 2D carbon-based materials for future energy storage and conversion device applications.

## Conflicts of interest

There are no conflicts to declare.

## Supplementary Material
